# A bias-reduced generalized estimating equation approach for proportional odds models with small-sample longitudinal ordinal data

**DOI:** 10.1186/s12874-024-02259-6

**Published:** 2024-06-28

**Authors:** Yukio Tada, Tosiya Sato

**Affiliations:** 1 Biostatistics Center, Shionogi & Co., Ltd., Osaka, Japan; 2 Department of Biostatistics, Kyoto University School of Public Health, Kyoto, Japan

**Keywords:** Bias reduction, Marginal model, Categorical data, Penalization, Firth’s adjustment

## Abstract

**Background:**

Longitudinal ordinal data are commonly analyzed using a marginal proportional odds model for relating ordinal outcomes to covariates in the biomedical and health sciences. The generalized estimating equation (GEE) consistently estimates the regression parameters of marginal models even if the working covariance structure is misspecified. For small-sample longitudinal binary data, recent studies have shown that the bias of regression parameters may result from the GEE and have addressed the issue by applying Firth’s adjustment for the likelihood score equation to the GEE as if generalized estimating functions were likelihood score functions. In this manuscript, for the proportional odds model for longitudinal ordinal data, the small-sample properties of the GEE were investigated, and a bias-reduced GEE (BR-GEE) was derived.

**Methods:**

By applying the adjusted function originally derived for the likelihood score function of the proportional odds model to the GEE, we produced the BR-GEE. We investigated the small-sample properties of both GEE and BR-GEE through simulation and applied them to a clinical study dataset.

**Results:**

In simulation studies, the BR-GEE had a bias closer to zero, smaller root mean square error than the GEE with coverage probability of confidence interval near or above the nominal level. The simulation also showed that BR-GEE maintained a type I error rate near or below the nominal level.

**Conclusions:**

For the analysis of longitudinal ordinal data involving a small number of subjects, the BR-GEE is advantageous for obtaining estimates of the regression parameters of marginal proportional odds models.

**Supplementary Information:**

The online version contains supplementary material available at 10.1186/s12874-024-02259-6.

## Introduction

Longitudinal ordinal data are frequently collected in biomedical and health science studies. In such a study, each subject is observed repeatedly over a period, and ordinal responses of interest at each observation are recorded. Because repeated responses from the same subject are usually correlated, a straightforward application of generalized linear models for a single response variable to longitudinal data is not appropriate. There are numerous approaches for extending generalized linear models to longitudinal data. One approach for extending generalized linear models to longitudinal data is a class of regression models where the model for the mean response at each observation does not incorporate dependence on any random effects or previous responses. The model is known as a marginal model. An alternative approach for accounting for the within-subject association is via the introduction of random effects. The model is known as the generalized linear mixed effects model. Due to the interpretation of their regression coefficients, the former are often referred to as “population-average models,” and the latter are referred to as “subject-specific models” [1]. Assuming a situation where the target of inference is the population-level summary described as a basis for comparison between treatment conditions in ICH E9 (R1), “Addendum on Estimands and Sensitivity Analysis in Clinical Trials” to the guideline on Statistical Principles for Clinical Trials [2], we focus on marginal models here. For estimation of the marginal model parameters, assumptions about the joint distribution of the responses are not necessary. The avoidance of distributional assumptions leads to a method of estimation known as the generalized estimating equation (GEE) [3]. An appealing property of the GEE estimator is that it is a consistent estimator even if the assumed model for the covariance among the repeated measures is not correctly specified. In addition, we can obtain valid variance estimate for regression coefficients by utilizing the empirical or sandwich estimator. The sandwich estimator possesses a notable property as it demonstrates robustness by providing valid variance estimate even when the working covariance among the repeated measures is not correct. The GEE requires only that the model for the mean response, the link function and the variance of each response be correct.

For binary response data, recent studies have investigated the small-sample property of GEE estimates of the regression parameter and the bias-reduced estimates obtained by applying Firth’s adjustment for the likelihood score equation [4] to the GEE as if generalized estimating functions were likelihood score functions [5–8]. Paul and Zhang [5] found that GEE estimates yielded biased estimates of the regression parameters when the sample size was small and that bias-reduced estimates improved bias and mean squared error (MSE) by simulation studies. Mondol and Rahman [6] showed that bias-reduced GEE (also referred to as penalized GEE) achieved convergence and provided finite estimates in the presence of separation. Geroldinger et al. [7] reported that the bias-reduced estimation improved convergence compared to the standard GEE with a similar or even better performance in terms of the accuracy of estimates. Gosho et al. [8] reviewed bias-reduced GEEs and modified covariance estimators and evaluated their performance in sparse binary data from small-sample longitudinal studies. In a study investigating marginal models in small samples, Bie et al. [9] reported that both GEE and marginalized multilevel models exhibit small-sample bias when correct correlation structure is adopted. Greenland [10] states that “the potential for small-sample bias in results from asymptotic procedures needs to be checked more routinely than is current practice.”

The proportional odds model is the commonly used model for relating ordinal outcome to covariates. The model is also referred to as the proportional odds version of the cumulative logit model [11]. The proportional odds model assumes that the association of each covariate with the outcome is represented by a single odds ratio. The purpose of this paper is to obtain the bias-reduced GEE (BR-GEE) estimates of the proportional odds model for longitudinal ordinal data and to investigate the small-sample properties of both the GEE and the BR-GEE through simulation. Kosmidis and Firth [12] derived general expressions for the adjusted score equations for the general class of multivariate models. Using the adjusted score equations exploited in Kosmidis and Firth [12] for the multivariate generalized linear model, Kosmidis [13] derived the adjusted score equation for the proportional odds model. We apply the adjusted likelihood score equation for the proportional odds model to the GEE to obtain the BR-GEE estimate as a solution to an adjusted estimating equation, which is induced by adding the adjustment function.

“Methods” section provides a brief summary of the BR-GEE estimates for longitudinal ordinal data. In “Simulation study” section, the results of the simulation study are presented and the small-sample properties of the GEE and the BR-GEE are investigated. In “Example” section, we apply the BR-GEE to the data of postoperative pain after laparoscopic cholecystectomy illustrated in Lumley [14]. A discussion of our findings follows in “Discussion” section.

## Methods

### Notation and GEE for the proportional odds model

Suppose a study includes $N$ subjects with ${n}_{i}$ repeated observations of the $K$ multinomial ordered categories for the *i*th subject, $i=1,\dots ,N$. For simplicity, we assume equal repetition, ${n}_{i}=n$. Let ${{\varvec{Y}}}_{it}$ represent responses as a vector ${{\varvec{Y}}}_{it}={\left({Y}_{it1},\dots ,{Y}_{itq}\right)}^{T}$ of $q=K-1$ dummy variables, where ${Y}_{itk}=1$ for the observation at occasion $t (t=1,\dots ,n)$ of subject $i (i=1,\dots ,N)$ falling in category $k (k=1,\dots ,q)$ and ${Y}_{itk}=0$ otherwise. The vector of marginal means or response probabilities of ${{\varvec{Y}}}_{it}$ is ${{\varvec{\pi}}}_{it}={\left({\pi }_{it1},\dots ,{\pi }_{itq}\right)}^{T}$. The proportional odds model links the cumulative probability ${\gamma }_{itk}={\pi }_{it1}+\dots +{\pi }_{itk}$ to a $p$-vector of covariates ${\mathbf{x}}_{it}$ via the following relationship:

$$\begin{array}{cc}\text{log}\left(\frac{{\gamma }_{itk}}{1-{\gamma }_{itk}}\right)={\beta }_{0k}+{\text{x}}_{it}^{T}{\varvec{\beta}}& \left(i=1,\dots ,N;t=1,\dots ,n;k=1,\dots ,q\right),\end{array}$$

where ${\varvec{\delta}}={\left({\beta }_{01},\dots ,{\beta }_{0q},{{\varvec{\beta}}}^{T}\right)}^{T}$ is a $\left(q+p\right)$-vector of model parameters with ${\beta }_{01}<\dots <{\beta }_{0q}$ and ${\varvec{\beta}}={\left({\beta }_{1},\dots ,{\beta }_{p}\right)}^{T}$. The linear term ${\eta }_{itk}={\beta }_{0k}+{\text{x}}_{it}^{T}{\varvec{\beta}}$ is determined by ${\eta }_{itk}={\sum }_{r=1}^{p+q}{\delta }_{r}{z}_{itkr}$, where ${z}_{itkr}$ is the $\left(k,r\right)$ th element of the $q\times \left(q+p\right)$ matrix,

$$\begin{array}{cc}{{\varvec{Z}}}_{it}=\left(\begin{array}{ccccc}1& 0& \cdots & 0& {\text{x}}_{it}^{T}\\ 0& 1& \cdots & 0& {\text{x}}_{it}^{T}\\ \vdots & \vdots & \ddots & \vdots & \vdots \\ 0& 0& \cdots & 1& {\text{x}}_{it}^{T}\end{array}\right)& \left(i=1,\dots ,N;t=1,\dots ,n\right)\end{array}.$$

Let ${{\varvec{Y}}}_{i}={\left({{\varvec{Y}}}_{i1}^{T},\dots ,{{\varvec{Y}}}_{in}^{T}\right)}^{T}$ and ${{\varvec{\pi}}}_{i}={\left({{\varvec{\pi}}}_{i1}^{T},\dots ,{{\varvec{\pi}}}_{in}^{T}\right)}^{T}$ denote responses and response probabilities, respectively, for subject $i$. Then, a GEE for consistent estimation of ${\varvec{\delta}}$ is given in the form,

$${\varvec{U}}\left({\varvec{\delta}},\alpha \right)=\sum_{i=1}^{N}{{\varvec{D}}}_{i}^{T}{{\varvec{V}}}_{i}^{-1}\left({{\varvec{Y}}}_{i}-{{\varvec{\pi}}}_{i}\right)=\bf{0},$$

where ${{\varvec{D}}}_{i}=D\left({{\varvec{\pi}}}_{i};{\varvec{\delta}}\right)$ is the $qn\times p$ Jacobian of ${{\varvec{\pi}}}_{i}$ with respect to ${\varvec{\updelta}}$ and ${{\varvec{V}}}_{i}={{\varvec{V}}}_{i}({\varvec{\delta}},\alpha )$ is a ‘working’ covariance matrix indexed by ${\varvec{\updelta}}$ and an association parameter $\alpha$. Further details on the GEE for ordinal data can be found in Agresti [11], Heagerty and Zeger [15], Fahrmeir and Pritscher [16], Lipsitz et al. [17], Liang et al. [18], and Williamson et al. [19].

Let ${{\varvec{V}}}_{itt}={\text{Var}}\left({{\varvec{Y}}}_{it}\right)=\text{diag}\left({{\varvec{\pi}}}_{it}\right)-{{\varvec{\pi}}}_{it}{{\varvec{\pi}}}_{it}^{T}$ denote the $q\times q$ multinomial covariance matrix for ${{\varvec{Y}}}_{it}$. For $t\ne {t}{\prime}$, ${{\varvec{V}}}_{it{t}{\prime}}={\text{Cov}}\left({{\varvec{Y}}}_{it},{{\varvec{Y}}}_{i{t}{\prime}}\right)$ is the covariance matrix between two different occasions in the same subject. The simplest model for the working covariance matrix is the independent model, ${{\varvec{V}}}_{it{t}{\prime}}=\bf{0}$. Lumley [14] described working covariance structures ${{\varvec{V}}}_{it{t}{\prime}}$ and computational methods for ordinal data based on the global odds ratio that allow the GEE to be used for smaller sets of ordinal data and with less effort expended on modeling associations. For each pair of categories $c$ and ${c}{\prime}$ of two ordinal responses ${R}_{it}$ and ${R}_{i{t}{\prime}}$ in the same subject, the global odds ratio is defined as

$$\begin{array}{cc}{\Psi }_{itc{t}{\prime}{c}{\prime}}=\frac{\text{Pr}\left({R}_{it}\le c,{R}_{i{t}{\prime}}\le {c}{\prime}\right)\text{Pr}\left({R}_{it}>c,{R}_{i{t}{\prime}}>{c}{\prime}\right)}{\text{Pr}\left({R}_{it}>c,{R}_{i{t}{\prime}}\le {c}{\prime}\right)\text{Pr}\left({R}_{it}\le c,{R}_{i{t}{\prime}}>{c}{\prime}\right)},& c,{c}{\prime}=1,\dots ,q.\end{array}$$

The covariance of ${Y}_{itc}$ and ${Y}_{i{t}{\prime}{c}{\prime}}$ is given by $\text{cov}\left({Y}_{itc}, {Y}_{i{t}{\prime}{c}{\prime}}\right)=E\left({Y}_{itc}{Y}_{i{t}{\prime}{c}{\prime}}\right)-E\left({Y}_{itc}\right)E\left({Y}_{i{t}{\prime}{c}{\prime}}\right)$, where the second term is the product of ${\pi }_{itc}$ and ${\pi }_{i{t}{\prime}{c}{\prime}}$. The first term $E\left({Y}_{itc}{Y}_{i{t}{\prime}{c}{\prime}}\right)$ can be expressed by the joint cumulative probabilities ${\gamma }_{itc{t}{\prime}{c}{\prime}}=\text{Pr}\left({R}_{it}\le c, {R}_{i{t}{\prime}}\le {c}{\prime}\right)$:

$$E\left({Y}_{itc}{Y}_{i{t}{\prime}{c}{\prime}}\right)=\left\{\begin{array}{cc}\begin{array}{c}{\gamma }_{itc{t}{\prime}{c}{\prime}}\\ {\gamma }_{itc{t}{\prime}{c}{\prime}}-{\gamma }_{itc{t}{\prime}\left({c}{\prime}-1\right)}\\ {\gamma }_{itc{t}{\prime}{c}{\prime}}-{\gamma }_{it\left(c-1\right){t}{\prime}{c}{\prime}}\\ {\gamma }_{itc{t}{\prime}{c}{\prime}}-{\gamma }_{itc{t}{\prime}\left({c}{\prime}-1\right)}-{\gamma }_{it\left(c-1\right){t}{\prime}{c}{\prime}}+{\gamma }_{it(c-1){t}{\prime}({c}{\prime}-1)}\end{array}& \begin{array}{c}c={c}{\prime}=1\\ c=1,{c}{\prime}>1\\ c>1,{c}{\prime}=1\\ c>1,{c}{\prime}>1\end{array}\end{array}\right.$$

The joint cumulative probabilities ${\gamma }_{itc{t}{\prime}{c}{\prime}}$ can be expressed in terms of global odds ratio and marginal cumulative probabilities:

1 $${\gamma }_{itc{t}{\prime}{c}{\prime}}=\frac{\kappa -\sqrt{{\kappa }^{2}-4{\Psi }_{itc{t}{\prime}{c}{\prime}}({\Psi }_{itc{t}{\prime}{c}{\prime}}-1){\gamma }_{itc}{\gamma }_{i{t}{\prime}{c}{\prime}}}}{2({\Psi }_{itc{t}{\prime}{c}{\prime}}-1)},$$

where $\kappa =1+({\gamma }_{itc}+{\gamma }_{i{t}{\prime}{c}{\prime}})({\Psi }_{itc{t}{\prime}{c}{\prime}}-1)$. A simple estimate of the crude global odds ratio is calculated by the weighted mean of the log odds ratios with weights inversely proportional to the variances for every possible pair of the two time points $\left(t,{t}{\prime}\right)$, where the odds ratios are computed based on the $2\times 2$ tables obtained from the ${q}^{2}$ ways of collapsing the row and column classifications into dichotomies for a $K\times K$ table of the variables ${R}_{it}$ and ${R}_{i{t}{\prime}}$ over all subjects. Hines [20] compared the various models for ${{\varvec{V}}}_{it{t}{\prime}}$ both in terms of efficiency and computational stability. She showed that the performance of Lumley’s crude global odds ratio method is satisfactory. Therefore, our approach is based on the working covariance structure of the independence or exchangeable model, where the crude global odds ratio is constant for all pairs of times.

### Bias-reduced GEE for the proportional odds model

Firth [4] showed that the solution of the following adjusted score equation results in an estimator that is free from the first-order term in the asymptotic expansion of the bias in the MLE of the regression parameter:

$${{\varvec{U}}}^{\boldsymbol{*}}\left({\varvec{\theta}}\right)={\varvec{U}}\left({\varvec{\theta}}\right)+{\varvec{A}}\left({\varvec{\theta}}\right)=\bf{0},$$

with ${\varvec{A}}\left({\varvec{\theta}}\right)=-{\varvec{I}}({\varvec{\theta}}){\varvec{b}}({\varvec{\theta}})$, where ${\varvec{\theta}}={({\theta }_{1},\dots ,{\theta }_{p})}^{T}$ is a $p$-vector of regression parameters from the generalized linear model, ${\varvec{U}}\left({\varvec{\theta}}\right)=\frac{\partial }{\partial{\varvec{\theta}}}l({\varvec{\theta}})=\bf{0}$ is the standard score equation based on the log-likelihood function $l({\varvec{\theta}})$, ${\varvec{I}}({\varvec{\theta}})$ is the expected information matrix for ${\varvec{\theta}}$, and ${\varvec{b}}({\varvec{\theta}})$ is the first term in the asymptotic expansion of the bias of the MLE.

Kosmidis and Firth [12] derived general expressions for the adjusted score functions for the general class of multivariate models, which include multivariate generalized linear models. Using the adjustment functions exploited in Kosmidis and Firth [12] for the score functions of the multivariate generalized linear model, Kosmidis [13] formulated the adjustment function of the score function of the proportional odds model. Considering that the GEE is equivalent to the generalized linear model score function under the identity working covariance structure, we treat ${\varvec{U}}\left({\varvec{\delta}},\alpha \right)$ as if it were a likelihood score function and apply the adjustment function in the score function to the GEE. Then, the adjustment function for the generalized estimating function for the proportional odds model is given by

$$\begin{array}{cc}{A}_{r}\left({\varvec{\delta}},\alpha \right)=\frac{1}{2}\sum\limits_{i=1}^{N}\sum\limits_{s=1}^{qn}{\text{tr}}\left[{{\varvec{Z}}}_{i}{{\varvec{F}}}^{-1}{{\varvec{Z}}}_{i}^{T}\left\{{\left(D\left({{\varvec{\pi}}}_{i};{{\varvec{\eta}}}_{i}\right){{\varvec{V}}}_{i}^{-1}\right)}_{s}\bigotimes{{\varvec{I}}}_{qn}\right\}{D}^{2}\left({{\varvec{\pi}}}_{i};{{\varvec{\eta}}}_{i}\right)\right]{z}_{isr}& (r=1,\dots ,q+p)\end{array},$$

where ${{\varvec{Z}}}_{i}={\left({{\varvec{Z}}}_{i1}^{T},\dots ,{{\varvec{Z}}}_{in}^{T}\right)}^{T}$, ${{\varvec{\eta}}}_{i}={({{\varvec{\eta}}}_{i1}^{T},\dots ,{{\varvec{\eta}}}_{in}^{T})}^{T}$ with elements of ${{\varvec{\eta}}}_{it}={({\eta }_{it1},\dots ,{\eta }_{itq})}^{T}$, $D\left({{\varvec{\pi}}}_{i};{{\varvec{\eta}}}_{i}\right)$ is the $qn\times qn$ Jacobian of ${{\varvec{\pi}}}_{i}$ with respect to ${{\varvec{\eta}}}_{i}$, ${\varvec{F}}={\sum }_{i=1}^{N}{(D\left({{\varvec{\pi}}}_{i};{{\varvec{\eta}}}_{i}\right){{\varvec{Z}}}_{i})}^{T}{{\varvec{V}}}_{i}^{-1}D\left({{\varvec{\pi}}}_{i};{{\varvec{\eta}}}_{i}\right){{\varvec{Z}}}_{i}$, ${D}^{2}\left({{\varvec{\pi}}}_{i};{{\varvec{\eta}}}_{i}\right)$ is the ${(qn)}^{2}\times qn$ matrix with the $s$th block as the Hessian of ${\pi }_{is}$ with respect to ${{\varvec{\eta}}}_{i} (s=1,\dots ,qn)$, ${{\varvec{I}}}_{qn}$ is the $qn\times qn$ identity matrix, ${z}_{isr}$ is the $\left(s,r\right)$ th element of ${{\varvec{Z}}}_{i}$, ${{\varvec{V}}}_{i}$ is the covariance matrix of the vector ${{\varvec{Y}}}_{i}$, and ${\left(D\left({{\varvec{\pi}}}_{i};{{\varvec{\eta}}}_{i}\right){{\varvec{V}}}_{i}^{-1}\right)}_{s}$ is the $s$th row of $D\left({{\varvec{\pi}}}_{i};{{\varvec{\eta}}}_{i}\right){{\varvec{V}}}_{i}^{-1}$. The matrix $D\left({{\varvec{\pi}}}_{i};{{\varvec{\eta}}}_{i}\right)$ is the block diagonal matrix whose $t$ th diagonal element is

$$\begin{array}{cc}{\Delta }_{it}=\left(\begin{array}{ccccc}{g}_{it1}& 0& \cdots & 0& 0\\ {-g}_{it1}& {g}_{it2}& \cdots & 0& 0\\ 0& {-g}_{it2}& \ddots & \vdots & \vdots \\ \vdots & \vdots & \ddots & {g}_{it,q-1}& 0\\ 0& 0& \cdots & -{g}_{it,q-1}& {g}_{itq}\end{array}\right)& \left(i=1,\dots ,N;t=1,\dots ,n\right)\end{array},$$

where ${g}_{itk}=g\left({\eta }_{itk}\right)$ with $g\left(\eta \right)=dG(\eta )/d\eta$ and $G\left(\eta \right)=\text{exp}(\eta )/(1+\text{exp}(\eta ))$; hence, ${g}_{itk}=G\left({\eta }_{itk}\right)(1-G\left({\eta }_{itk}\right))={\gamma }_{ikt}\left(1-{\gamma }_{ikt}\right)$.

The matrix ${D}^{2}\left({{\varvec{\pi}}}_{i};{{\varvec{\eta}}}_{i}\right)$ is the ${(qn)}^{2}\times qn$ matrix with the $\left(s,u\right)$ th element given by:

$$\begin{array}{cc}{c}_{su}=\left\{\begin{array}{c}{\gamma }_{itk}\left(1-{\gamma }_{itk}\right)\left(1-2{\gamma }_{itk}\right),\\ \begin{array}{c}-{\gamma }_{itk}\left(1-{\gamma }_{itk}\right)\left(1-2{\gamma }_{itk}\right)\\ 0,\end{array}\end{array}\right.& \begin{array}{l}s=\left(v-1\right)\left(nq+1\right)+1, u=v\\ s=w(nq+1)+1,u=w\\ otherwise\end{array}\end{array}$$

where $$v=\left(t-1\right)q+k \left(t=1,\dots ,n;k=1,\dots ,q\right)$$ and $$w=\left(t-1\right)q+k \left(t=1,\dots ,n;k=1,\dots ,q-1\right)$$.

The $r$ th element of the bias-reduced generalized estimating function is ${U}_{r}^{*}\left({\varvec{\delta}},\alpha \right)={U}_{r}\left({\varvec{\delta}},\alpha \right)+{A}_{r}\left({\varvec{\delta}},\alpha \right)$. The BR-GEE estimate $\widetilde{{\varvec{\delta}}}$ is such that ${U}_{r}^{*}\left(\widetilde{{\varvec{\delta}}},\widetilde{\alpha }\right)=0$ for every $r=1,\dots ,q+p$, where $\widetilde{\alpha }$ is the estimate of global odds ratio. $\widetilde{{\varvec{\delta}}}$ can be estimated using an iterative method described below:

Step 1: Choose an initial value ${\widetilde{{\varvec{\delta}}}}^{0}$ of ${\varvec{\delta}}$.

Step 2: If the working covariance structure is exchangeable, we calculate the log (crude) global odds ratio $\widetilde{\alpha }$ [14].

Step 3: Given ${\widetilde{{\varvec{\delta}}}}^{(t)}$ at the *t*th iteration and $\widetilde{\alpha }$ (unnecessary if the working covariance structure is independent), the covariance matrix is estimated from the global odds ratio and the predicted cumulative probabilities [14].

Step 4: Given the working covariance matrix ${{\varvec{V}}}_{i}^{-1}\left({\widetilde{{\varvec{\delta}}}}^{(t)},\widetilde{\alpha }\right)$, the current estimate ${\widetilde{{\varvec{\delta}}}}^{(t)}$ is updated according to the adjusted GEE using the Newton‒Raphson method given by

$${\widetilde{{\varvec{\delta}}}}^{\left(t+1\right)}={\widetilde{{\varvec{\delta}}}}^{\left(t\right)}+{\left.{{\varvec{F}}\left({\varvec{\delta}},\widetilde{\alpha }\right)}^{-1}\right|}_{{\varvec{\delta}}={\widetilde{{\varvec{\delta}}}}^{\left(t\right)}}{\left.{{\varvec{U}}}^{\boldsymbol{*}}\left({\varvec{\delta}},\widetilde{\alpha }\right)\right|}_{{\varvec{\delta}}={\widetilde{{\varvec{\delta}}}}^{\left(t\right)}},$$

with

$$F\left({\varvec{\delta}},\alpha \right)=\sum_{i=1}^{N}{{\varvec{D}}}_{i}^{T}{{\varvec{V}}}_{i}^{-1}{{\varvec{D}}}_{i}.$$

Step 5: Iterate steps 3 and 4 until a desired convergence criterion is satisfied (for example, $\text{max}\left|{\widetilde{{\varvec{\delta}}}}^{(t+1)}-{\widetilde{{\varvec{\delta}}}}^{(t)}\right|\le 0.0001$). At convergence, the estimate of ${\varvec{\delta}}$ is denoted by $\widetilde{{\varvec{\delta}}}$ and referred to as the BR-GEE estimate.

The consistent estimate of the covariance matrix of the GEE regression parameter estimates is given by the sandwich estimator of Liang and Zeger [3],

$${{{\varvec{\Sigma}}}_{{\varvec{G}}{\varvec{E}}{\varvec{E}}}=\left(\sum_{i=1}^{N}{{\varvec{D}}}_{i}^{T}{{\varvec{V}}}_{i}^{-1}{{\varvec{D}}}_{i}\right)}^{-1}\left(\sum_{i=1}^{N}{{\varvec{D}}}_{i}^{T}{{\varvec{V}}}_{i}^{-1}{\text{Cov}}({{\varvec{Y}}}_{i}){{\varvec{V}}}_{i}^{-1}{{\varvec{D}}}_{i}\right){\left(\sum_{i=1}^{N}{{\varvec{D}}}_{i}^{T}{{\varvec{V}}}_{i}^{-1}{{\varvec{D}}}_{i}\right)}^{-1},$$

where ${\widehat{{\varvec{r}}}}_{i}{\widehat{{\varvec{r}}}}_{i}^{T}=({{\varvec{Y}}}_{i}-{\widehat{{\varvec{\pi}}}}_{i}){({{\varvec{Y}}}_{i}-{\widehat{{\varvec{\pi}}}}_{i})}^{T}$ is used to estimate ${\text{Cov}}({{\varvec{Y}}}_{i})$.

In this manuscript, we calculate the covariance matrix for the BR-GEE derived from the estimating function ${{\varvec{U}}}^{\boldsymbol{*}}\left({\varvec{\delta}},\alpha \right)={\varvec{U}}\left({\varvec{\delta}},\alpha \right)+{\varvec{A}}\left({\varvec{\delta}},\alpha \right)$,

$${{{\varvec{\Sigma}}}_{{\varvec{B}}{\varvec{R}}-{\varvec{G}}{\varvec{E}}{\varvec{E}}}=\left(\sum_{i=1}^{N}{{\varvec{D}}}_{i}^{T}{{\varvec{V}}}_{i}^{-1}{{\varvec{D}}}_{i}+{\varvec{A}}{{\varvec{A}}}^{{\varvec{T}}}\right)}^{-1}{\varvec{M}}{\left(\sum_{i=1}^{N}{{\varvec{D}}}_{i}^{T}{{\varvec{V}}}_{i}^{-1}{{\varvec{D}}}_{i}+{\varvec{A}}{{\varvec{A}}}^{{\varvec{T}}}\right)}^{-1},$$

with

$${\varvec{M}}=\left(\sum_{i=1}^{N}{{\varvec{D}}}_{i}^{T}{{\varvec{V}}}_{i}^{-1}{\widehat{{\varvec{r}}}}_{i}{\widehat{{\varvec{r}}}}_{i}^{T}{{\varvec{V}}}_{i}^{-1}{{\varvec{D}}}_{i}+{\varvec{A}}{{\varvec{A}}}^{{\varvec{T}}}\right).$$

Mancl and DeRouen [21] described that the bias-corrected covariance estimator used with the critical value based on the F-distribution instead of chi-square produced proper test sizes. Therefore, in the following, we used the critical value of the t-distribution with the number of the sample size minus the number of coefficients in the regression model as the degree of freedom to compute the confidence interval and the statistical significance.

When the sample size is small, the sandwich covariance matrix is expected to underestimate the covariance matrix. To reduce the small-sample bias, several modified variance estimators have been developed [22]. In this manuscript, in addition to ${{\varvec{\Sigma}}}_{{\varvec{G}}{\varvec{E}}{\varvec{E}}}$ and ${{\varvec{\Sigma}}}_{{\varvec{B}}{\varvec{R}}-{\varvec{G}}{\varvec{E}}{\varvec{E}}}$**,** we used the bias-corrected covariance estimates of Mancl and DeRouen [21] obtained by substituting the GEE and BR-GEE estimates into the following equation in order to calculate the 95% confidence interval,

$${{\varvec{\Sigma}}}_{{\varvec{M}}{\varvec{D}}}={\left(\sum_{i=1}^{N}{{\varvec{D}}}_{i}^{T}{{\varvec{V}}}_{i}^{-1}{{\varvec{D}}}_{i}\right)}^{-1}{{\varvec{M}}}_{{\varvec{M}}{\varvec{D}}}{\left(\sum_{i=1}^{N}{{\varvec{D}}}_{i}^{T}{{\varvec{V}}}_{i}^{-1}{{\varvec{D}}}_{i}\right)}^{-1}.$$

with

$${{\varvec{M}}}_{{\varvec{M}}{\varvec{D}}}=\left(\sum_{i=1}^{N}{{\varvec{D}}}_{i}^{T}{{\varvec{V}}}_{i}^{-1}({{\varvec{I}}}_{qn}-{{\varvec{H}}}_{ii}){\widehat{{\varvec{r}}}}_{i}{\widehat{{\varvec{r}}}}_{i}^{T}{({{\varvec{I}}}_{qn}-{{\varvec{H}}}_{ii})}^{T}{{\varvec{V}}}_{i}^{-1}{{\varvec{D}}}_{i}\right),$$

where ${{\varvec{I}}}_{qn}$ is the $qn\times qn$ identity matrix and ${{\varvec{H}}}_{ii}$ is the matrix calculated by

$${{\varvec{H}}}_{ii}={{{\varvec{D}}}_{i}\left(\sum_{i=1}^{N}{{\varvec{D}}}_{i}^{T}{{\varvec{V}}}_{i}^{-1}{{\varvec{D}}}_{i}\right)}^{-1}{{\varvec{D}}}_{i}^{T}{{\varvec{V}}}_{i}^{-1}.$$

## Simulation study

In this section, we conducted a limited simulation study to investigate the small-sample properties of both the standard GEE (GEE) and the proposed bias-reduced GEE (BR-GEE) estimation of the regression parameters in a marginal model for correlated ordinal data. We assumed a randomized clinical trial with a parallel group design, where each subject was assigned to either the treatment group or the control group. Balanced correlated ordinal data with $K=3$ of the total number of response categories and with observations over $n=4$ time occasions were generated using the algorithm given in Ibrahim et al. [23]. They used the Goodman and Kruskal $\Gamma$ coefficient as a measurement of the association for the responses of each subject. We modified the algorithm to specify the association of the within-subject observation by using the global odds ratio. Specifically, we modified the macro developed by Ibrahim et al. [23] to calculate the joint probabilities of responses between two time points, which are used to generate correlated ordinal data, by substituting the global odds ratio and marginal cumulative probability based on the marginal model into expression (1). For the exchangeable correlation, we used a common $\text{exp}\left(\alpha \right)$ as global odds ratio for all pairs of time points, while for AR-type correlation, we used $\text{exp}\left(\alpha /\tau \right)$ as global odds ratio based on the time interval $\tau$ for each pair of time points. We used a proportional odds marginal model both to simulate and to fit the data. We considered the following proportional odds model for the $i$ th subject measured at the $t$ th occasion ($i=1,\dots ,N;t=1,\dots ,4$):

2 $$\text{logit}\left({\gamma }_{itk}\right)={\beta }_{0k}+{\beta }_{1}{\text{Trt}}_{i}+\sum_{s=1}^{3}\left\{{\beta }_{s+1}I\left({\text{Time}}_{it}=s\right)+{\beta }_{s+4}{\text{Trt}}_{i}\times I\left({\text{Time}}_{it}=s\right)\right\}\begin{array}{cc}& (k=\text{1,2})\end{array} ,$$

where ${\gamma }_{itk}$ is the cumulative probability, ${\text{Trt}}_{i}$ is a treatment assignment variable coded as 1 for the first half of the subjects or 0 for the second half of the subjects, and ${\text{Time}}_{it}$ is an occasion coded as 1, 2, 3 and 4. We conducted simulations for four different sample sizes: $N=20, 30, 40, 50$. We let the $(q+p)$-vector parameter set used with regression model (2) be ${\varvec{\delta}}={\left({\beta }_{01},{\beta }_{02},{\beta }_{1},{\beta }_{2},{\beta }_{3},{\beta }_{4},{\beta }_{5},{\beta }_{6},{\beta }_{7}\right)}^{T}={\left(-0.1, 1, 1.2, -0.9, -0.6, -0.3, -0.3, -0.2, -0.1\right)}^{T}$ to evaluate the performance under the alternative hypothesis (Scenario 1). In addition, we let ${\varvec{\delta}}={\left(1.1, 2.2, 0, -2.1, -1.4, -0.7, 0, 0, 0\right)}^{T}$ to evaluate the empirical type I error rate (Scenario 2). For this set of simulations, the marginal response probabilities of the treatment group at the last time point are $\left(\text{0.75,0.15,0.10}\right)$. The covariance structure used to generate data was an exchangeable structure with the log global odds ratio of $\alpha$ as $1, 1.5, 2$ and an autoregressive-type (AR-type) structure with the log global odds ratio of $\alpha /\left|t-{t}{\prime}\right| (t\ne {t}{\prime})$ for each $\alpha$ as $1, 1.5, 2$. For each parameter configuration, 5,000 simulation replications were performed.

For each scenario, we fit the correct marginal mean model, and the covariate coefficients were estimated by the GEE and the BR-GEE with the independent and exchangeable in terms of the crude global odds ratio suggested in Lumley [14] as the working covariance structure. The algorithm convergence criterion was less than or equal to 0.0001, and the maximum iterations allowed were set to 50. The standard errors of the GEE and the BR-GEE estimated regression coefficients were estimated by substituting each parameter estimate into ${{\varvec{\Sigma}}}_{{\varvec{G}}{\varvec{E}}{\varvec{E}}}$ and ${{\varvec{\Sigma}}}_{{\varvec{B}}{\varvec{R}}-{\varvec{G}}{\varvec{E}}{\varvec{E}}}$, respectively. We defined a model fit as having a convergence problem if the algorithm convergence criterion was not met or if the maximum of the absolute value of the estimated regression parameters was above 10. There were a small number of datasets where the diagonal elements of the sandwich variance estimate were negative when there was a convergence problem. At that time, we replaced it with the model-based variance to evaluate the performance measures. We focused on the results of the coefficient ${\beta }_{1}$, which represents the treatment effect at the last time point. In this simulation study, we defined the bias as $\text{bias}\left({\widehat{\beta }}_{1}\right)={\widehat{\beta }}_{1}-{\beta }_{1}$, where ${\widehat{\beta }}_{1}={\sum }_{l=1}^{{L}_{s}}{\widehat{\beta }}_{1l}/{L}_{s}$, ${\widehat{\beta }}_{1l}$ is the estimate of ${\beta }_{1}$ for the $l$ th replication, and ${L}_{s}$ is the number of runs in which the regression parameter was estimated. The root mean square error (RMSE) was defined by $\text{RMSE}\left({\widehat{\beta }}_{1}\right)=\sqrt{{\sum }_{l=1}^{{L}_{s}}{({\widehat{\beta }}_{1l}-{\beta }_{1})}^{2}/{L}_{s}}$. Using the standard error estimate, we constructed 95% confidence intervals by multiplying the standard error with the 2.5th percentile and 97.5th percentile of the t-distribution. Then, we defined the coverage probability of 95% confidence intervals for the parameter ${\beta }_{1}$ to be the proportion of the number of 95% confidence intervals that contain the true parameter value to the total number of runs where the regression parameter was estimated. Furthermore, the empirical type I error rate was defined as the proportion of times $\left|{\widehat{\beta }}_{1l}/{\text{SE}}({\widehat{\beta }}_{1l})\right|\ge {t}_{0.975}$ for the null hypothesis, where ${t}_{0.975}$ is the 97.5th percentile of the t-distribution.

In addition, the bias, RMSE and coverage for the specified scenarios with $\left(K,n\right)=\left(\text{3,6}\right), \left(\text{4,4}\right), \left(\text{4,6}\right)$ were investigated. And, for each scenario, coverage probability of the 95% confidence interval based on the Mancl and DeRouen’s bias-corrected covariance estimates were calculated in addition to that based on ${{\varvec{\Sigma}}}_{{\varvec{G}}{\varvec{E}}{\varvec{E}}}$ and ${{\varvec{\Sigma}}}_{{\varvec{B}}{\varvec{R}}-{\varvec{G}}{\varvec{E}}{\varvec{E}}}$.

### Results for the convergence

First, we report the percentage of simulation replications in which there was evidence of quasicomplete separation as well as the percentage of simulation sets where the model had convergence problems for Scenario 1 and Scenario 2 (see Table S1 for the results with true exchangeable covariance structure (Scenario 1), Table S2 for the results with true exchangeable covariance structure (Scenario 2), Table S3 for the results with true AR-type covariance structure (Scenario 1), and Table S4 for the results with true AR-type covariance structure (Scenario 2) in Additional file). We defined that the simulation datasets had evidence of quasicomplete separation when all categories but one level of 1 or $K$ of the outcome for either treatment group were zero at a time point, such as Table 1. The model fit by the GEE had the convergence problem in the presence of quasicomplete separation. For instance, the percentage of datasets where GEE estimation had a convergence problem fell within the range 7.30–8.06 when $N=20$, 1.56–1.66 when $N=30$, 0.28–0.40 when $N=40$, and 0.06–0.08 when $N=50$ for Scenario 1 with true exchangeable covariance structure. In contrast, it was 0.00% for all settings for the BR-GEE. The performance of the estimates of ${\beta }_{1}$ was similar for both true covariance structures. Therefore, here, we present the results of the estimates of ${\beta }_{1}$ in the true exchangeable covariance structure only. See Additional file for the results in the AR-type covariance structure.

**Table 1:** Example of quasicomplete separation due to treatment (Trt) against outcome $Y$ (*N* = 40)

		Quasicomplete separation		
		$$Y$$		
		1	2	3
Trt	1	20	0	0
	0	5	5	10

### Results for the performance under the alternative hypothesis (Scenario 1)

In this section, we first report the distribution of the estimates. Then, we summarize the results for the simulation in terms of bias, RMSE, and coverage probability of the 95% confidence interval.

**Fig. 1:**
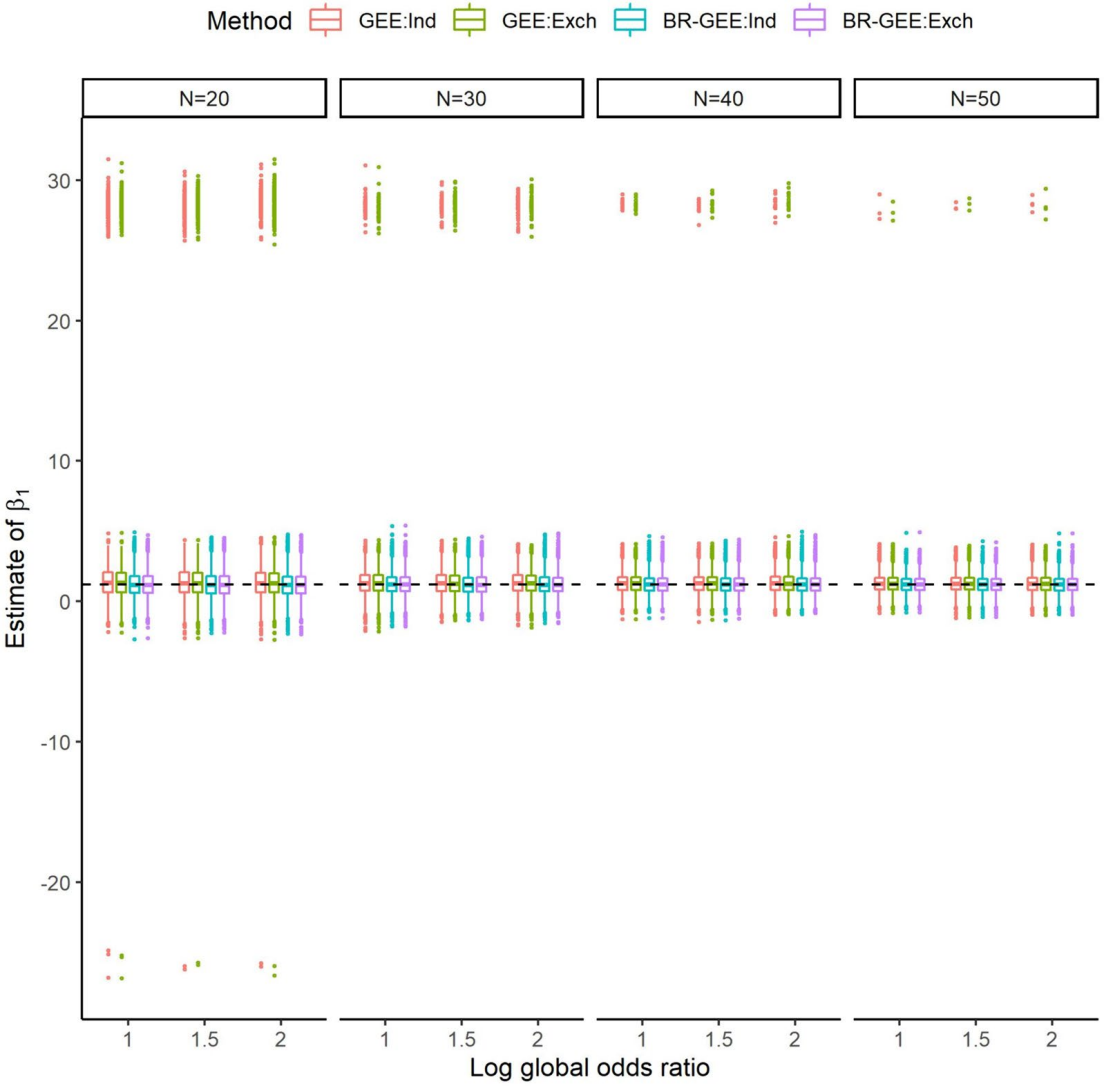
Box and whisker plot for estimates of ${\beta }_{1}$ with true exchangeable covariance structure (Scenario 1). GEE:Ind, generalized estimating equation with working independent covariance structure, GEE:Exch, generalized estimating equation with working exchangeable covariance structure, BR-GEE:Ind, bias-reduced generalized estimating equation with working independent covariance structure, BR-GEE:Exch, generalized estimating equation with working exchangeable covariance structure

Figure 1 shows the box and whisker plot for estimates of ${\beta }_{1}$ with an exchangeable (correctly specified) and an independent (misspecified) working covariance for the true exchangeable covariance structure. The distribution of the GEE estimates had substantially large outliers for datasets with evidence of quasicomplete separation. On the other hand, the BR-GEE did not have such outliers, regardless of the value of the global odds ratio and the working covariance structure for each sample size ($N\le 50$). See Fig. S1 for the results in the AR-type covariance structure.

**Fig. 2:**
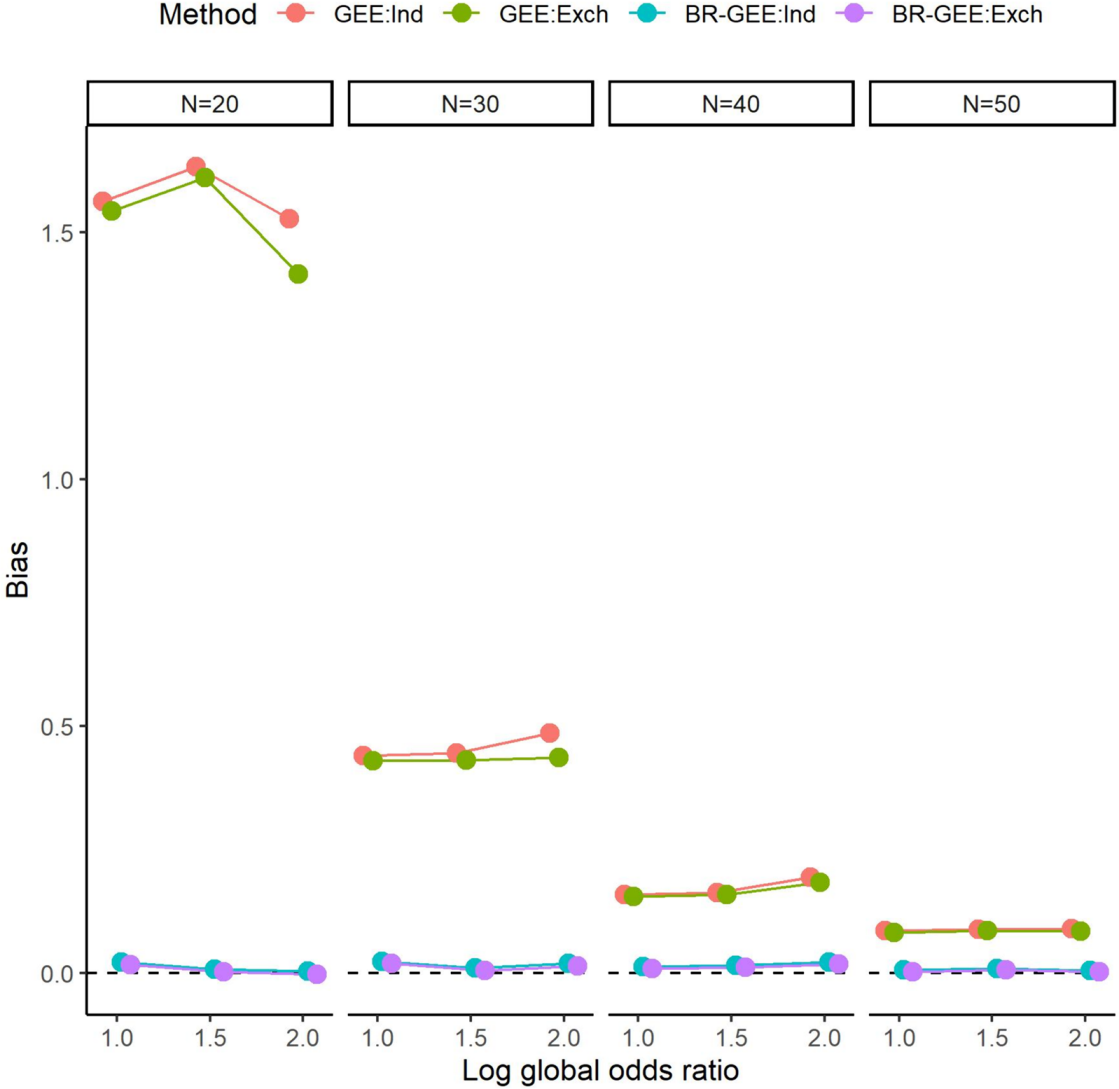
Bias associated with estimates of ${\beta }_{1}$ with true exchangeable covariance structure (Scenario 1). GEE:Ind, generalized estimating equation with working independent covariance structure, GEE:Exch, generalized estimating equation with working exchangeable covariance structure, BR-GEE:Ind, bias-reduced generalized estimating equation with working independent covariance structure, BR-GEE:Exch, generalized estimating equation with working exchangeable covariance structure

Figure 2 shows the bias of the estimates of ${\beta }_{1}$. Although it decreased as the sample size increased, bias of the GEE estimates still existed when $N=50$. On the other hand, the bias of the BR-GEE estimates was close to zero regardless of the value of the global odds ratio and the working covariance structure for each sample size ($N\le 50$). See Fig. S2 for the results in the AR-type covariance structure.

**Fig. 3:**
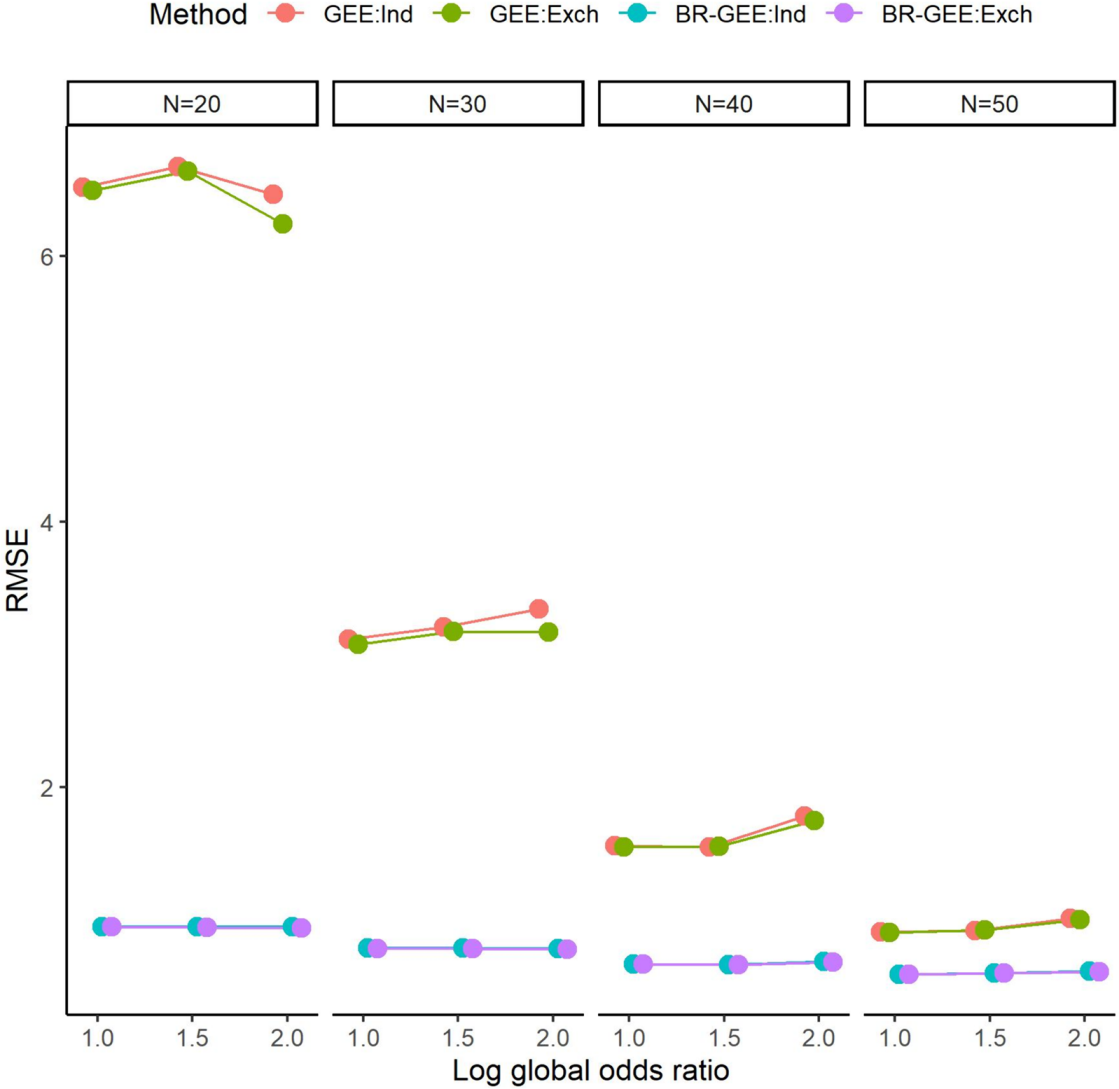
RMSE associated with estimates of ${\beta }_{1}$ with true exchangeable covariance structure (Scenario 1). GEE:Ind, generalized estimating equation with working independent covariance structure, GEE:Exch, generalized estimating equation with working exchangeable covariance structure, BR-GEE:Ind, bias-reduced generalized estimating equation with working independent covariance structure, BR-GEE:Exch, generalized estimating equation with working exchangeable covariance structure

Figure 3 shows the RMSE of the estimates of ${\beta }_{1}$. The RMSE of the BR-GEE estimates was smaller than that of the GEE estimates regardless of the value of the global odds ratio and the working covariance structure for each sample size ($N\le 50$). See Fig. S3 for the results in the AR-type covariance structure.

**Fig. 4:**
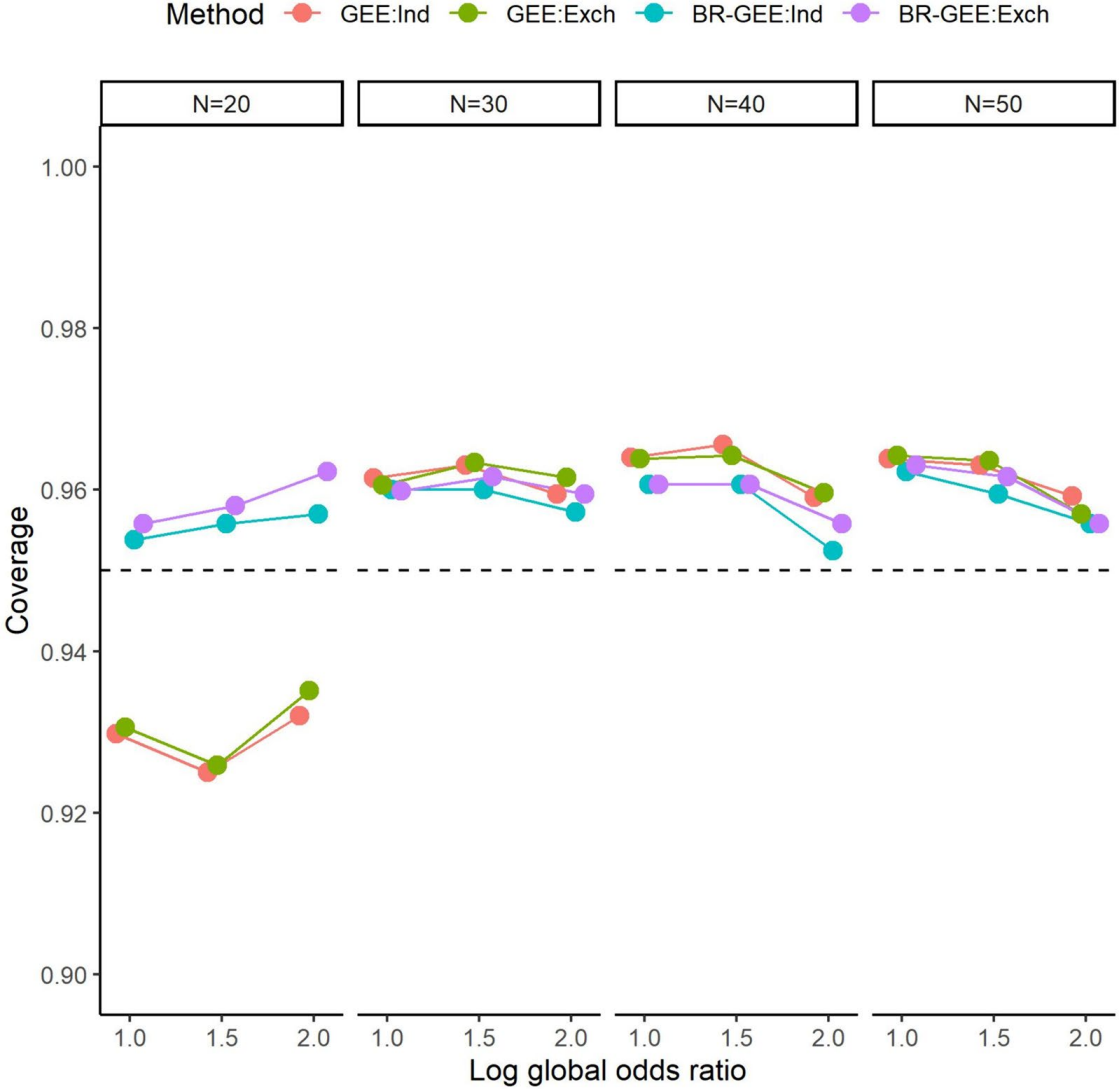
95% confidence interval coverage for estimates of ${\beta }_{1}$ with true exchangeable covariance structure (Scenario 1). GEE:Ind, generalized estimating equation with working independent covariance structure, GEE:Exch, generalized estimating equation with working exchangeable covariance structure, BR-GEE:Ind, bias-reduced generalized estimating equation with working independent covariance structure, BR-GEE:Exch, generalized estimating equation with working exchangeable covariance structure

Figure 4 shows the coverage probabilities of the 95% confidence interval of ${\beta }_{1}$. For $N$=20, the coverage probability of the 95% confidence interval based on the GEE was below the nominal level of 95%; on the other hand, that based on the BR-GEE was close to 95%. In general, as the number of subjects increased ($N\ge$ 30), the coverage probabilities of both methods became similar and slightly above 95%. See Fig. S4 for the results in the AR-type covariance structure. When the 95% confidence interval based on the BR-GEE was estimated by substituting the parameter estimate into ${{\varvec{\Sigma}}}_{{\varvec{G}}{\varvec{E}}{\varvec{E}}}$ instead of ${{\varvec{\Sigma}}}_{{\varvec{B}}{\varvec{R}}-{\varvec{G}}{\varvec{E}}{\varvec{E}}}$, undercoverage of the 95% confidence interval based on the BR-GEE was observed for $N$=20 but not for $N\ge$ 30. See Fig. S6 for the results in the exchangeable covariance structure, and Fig. S7 for those in the AR-type covariance structure.

The bias and RMSE for the scenarios with $\left(K,n\right)=\left(\text{3,6}\right), \left(\text{4,4}\right), \left(\text{4,6}\right)$ were similar to the results when $K=3$ and $n=4$. The differences in coverage probabilities between GEE and BR-GEE exhibited similar characteristics for $\left(K,n\right)=\left(\text{3,4}\right)\text{ and }\left(3,6\right)$. Specifically, when $N=20$, the coverage probabilities based on the GEE fell below the nominal level while those based on the BR-GEE exceeded it. However, when $K=4$, such differences in coverage probabilities between GEE and BR-GEE were not observed. See Fig. S10 to Fig. S27 in Additional file. The coverage probability of the 95% confidence interval based on the Mancl and DeRouen’s bias-corrected covariance estimates calculated by substituting the BR-GEE estimates generally remained above the nominal confidence level, similar to that of the 95% confidence interval based on ${{\varvec{\Sigma}}}_{{\varvec{B}}{\varvec{R}}-{\varvec{G}}{\varvec{E}}{\varvec{E}}}$. See Table. S5 to Table. S12 in Additional file.

### Results for the type I error rate under the null hypothesis (Scenario 2)

**Fig. 5:**
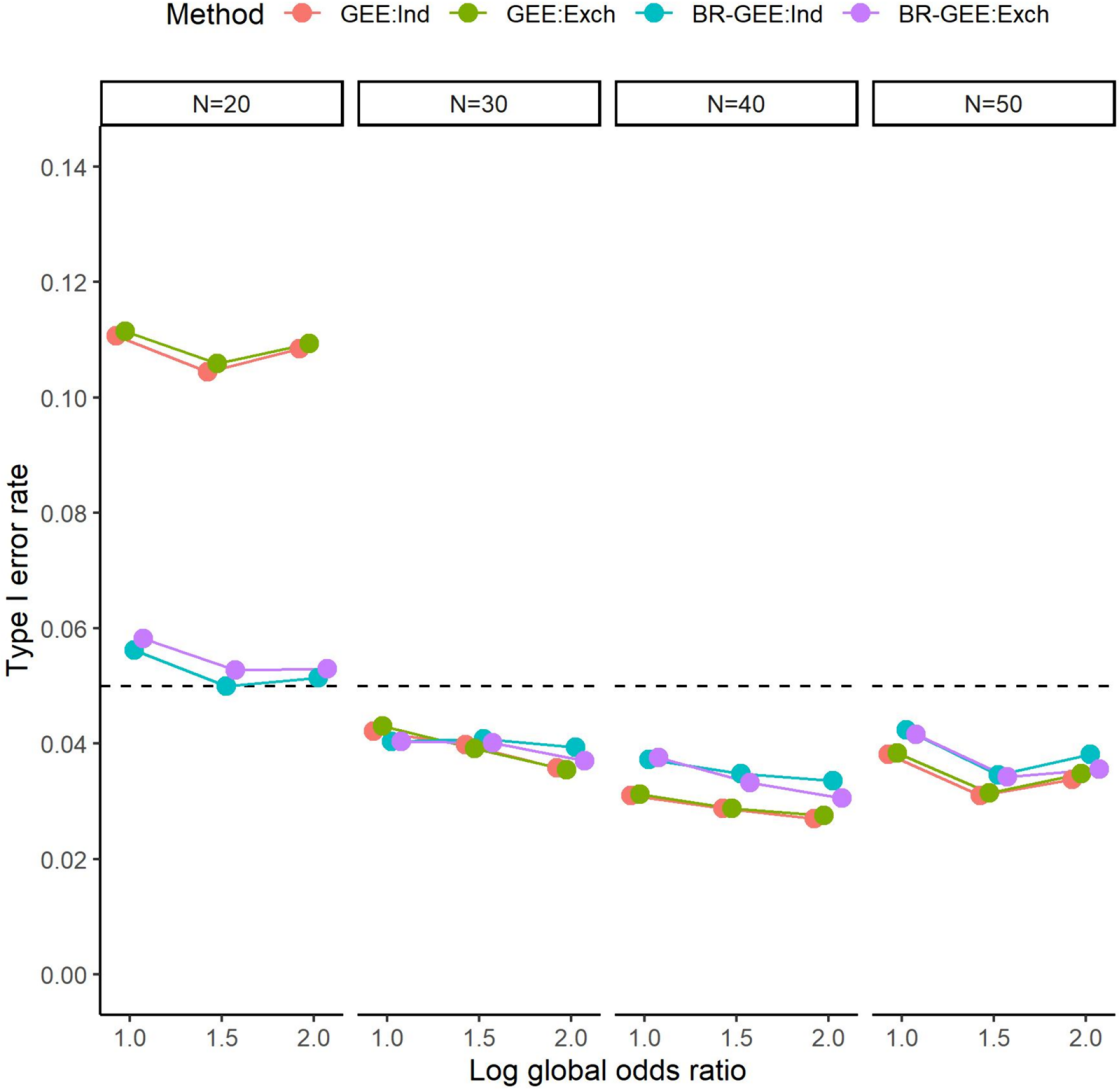
Type I error rate of t-test of ${H}_{0}:{\beta }_{1}=0$ with true exchangeable covariance structure (Scenario 2). GEE:Ind, generalized estimating equation with working independent covariance structure, GEE:Exch, generalized estimating equation with working exchangeable covariance structure, BR-GEE:Ind, bias-reduced generalized estimating equation with working independent covariance structure, BR-GEE:Exch, generalized estimating equation with working exchangeable covariance structure

Figure 5 presents the empirical type I error rate of the t-test under the null hypothesis ${H}_{0}:{\beta }_{1}=0$ resulting from the use of the GEE or the BR-GEE. For $N$=20, although the type I error rate for the GEE was somewhat inflated, that of the BR-GEE was approximately 0.05. In general, as the number of subjects increased ($N\ge$ 30), the type I error rates of both methods became similar and slightly conservative. See Fig. S5 for the results in the AR-type covariance structure. For the empirical type I error rate of the t-test under the null hypothesis, ${H}_{0}:{\beta }_{1}=0$, when the standard error for the BR-GEE as well as the GEE was estimated using the standard sandwich variance estimator, inflation of the type I error rate was observed for $N$=20 but not for $N\ge$ 30. See Fig. S8 for the results in the exchangeable covariance structure, and Fig. S9 for those in the AR-type covariance structure.

## Example

In this section, we applied the proposed method to data from a randomized clinical trial of patients with postoperative pain after laparoscopic cholecystectomy. One of the aims of the study was to determine whether the use of an abdominal suction drain reduces shoulder tip pain after laparoscopic surgery. Patients rated their shoulder pain levels on a visual analog scale in the morning and afternoon of the first 3 days after the operation. The dataset from Lumley [14] contained 41 patients (22 patients in the active treatment group and 19 patients in the control group) with 6 time points and 5 ordered pain score categories per patient. The pain in the control group peaked on the afternoon of day 2 [24]. Therefore, in this manuscript, we analyzed the data of 4 time points (Day 1 (am), Day 1 (pm), Day 2 (am), and Day 2 (pm)) of all 6 time points and estimated the treatment effect at Day 2 (pm). The time points for each morning and afternoon from Day 1 to Day 2 are designated as time = 1 to time = 4 in sequential order. To estimate the treatment effect at the last time point, we fit the marginal model of the proportional odds model with 9 covariates: 8 binary indicators (treatment, sex, time point, interaction of treatment and time point) and one continuous variable (age),

$$\text{logit}\left({\gamma }_{itk}\right)={\beta }_{0k}+{\beta }_{1}I\left({\text{Trt}}_{i}=1\right)+{\beta }_{2}I\left({\text{Sex}}_{i}=1\right)+{\beta }_{3}{\text{Age}}_{i}+\sum_{s=1}^{3}\left\{{\beta }_{s+3}I\left({\text{Time}}_{it}=s\right)+{\beta }_{s+6}I\left({\text{Trt}}_{i}=1\right)\times I\left({\text{Time}}_{it}=s\right)\right\}\begin{array}{cc}& (k=1\text{ to }4)\end{array},$$

where ${\text{Sex}}_{i}=1$ denotes that the subject is male, ${\text{Trt}}_{i}=1$ if the subject receives the active treatment, and 0 otherwise. For this model, we estimated the regression parameter by using the GEE and the BR-GEE, assuming an independent and exchangeable working covariance structure. In addition, we calculated the 95% confidence interval by using the t-distribution. In this model, the regression parameter ${\beta }_{1}$ represents the treatment effect at the last time point $\left(\text{Time}=4\right)$.

The odds ratio of favorable response comparing the active treatment group with the control group and the 95% confidence intervals by using the GEE with independent working covariance structure, the GEE with exchangeable working covariance structure, the BR-GEE with independent working covariance structure, and the BR-GEE with exchangeable working covariance structure were 14.0 (3.6, 54.5), 12.8 (3.5, 47.0), 12.1 (3.6, 41.1) and 10.6 (3.2, 34.8), respectively. Each of the odds ratios can be interpreted as a common odds ratio derived from 4 logistic regression models for each possible binary dichotomization (1 vs. 2,3,4,5; 1,2 vs. 3,4,5; 1,2,3 vs. 4,5; 1,2,3,4 vs. 5) of the outcome. Overall, the estimate was smaller for the BR-GEE than for the GEE. And the results from any methods suggested that subjects with the active treatment were likely to have a more favorable response when compared to subjects with the control treatment.

## Discussion

In this paper, we have investigated a BR-GEE to obtain GEE estimates of the proportional odds model for longitudinal ordinal data. The BR-GEE was derived by applying adjusted score equations for the proportional odds model in Kosmidis [13] to the GEE as if it were equivalent to the likelihood score equation. A similar approach for small-sample bias reduction in the GEE estimate for clustered binary data was investigated in recent studies (Paul and Zhang [5], Mondol and Rahman [6], Geroldinger [7], Gosho et al. [8]).

In the simulations that are reported here, the BR-GEE performs better than the standard GEE for small-sample by providing finite estimates in the presence of quasicomplete separation. This finding is consistent with that of Mondol and Rahman [6].

With the above consideration, in the estimation of the regression parameter when the proportional odds model is applied to longitudinal ordinal data in a small-sample size of 50 or less, it is advantageous to apply the BR-GEE instead of the standard GEE. However, these findings are subject to the following limitations. First, our simulation setting is limited. In fact, we did not consider simulations with extreme parameter settings where datasets with no observations for a specific category throughout all time points are generated. Such settings would lead to conditional performance evaluation, and therefore we decided to avoid such configurations in this study. Second, we assumed we had complete data, so further study may be required to evaluate the properties of the BR-GEE when there are missing data. In addition, the bias-corrected covariance estimator for GEE with longitudinal ordinal data may also be in need of further investigation. The bias correction methods for the variance–covariance matrix in small-sample GEE estimation have primarily been evaluated assuming binary or count response data. Considering ordinal response data, we believe that new insights can be obtained by applying the previously proposed methods as well as the method proposed in this study and conducting a detailed evaluation of operational characteristics. As alternative models when proportional odds model is not appropriate, we have other types of logit including the adjacent-categories or continuation-ratio. Furthermore, for applying the stereotype model to longitudinal data, estimation methods using GEE was considered [25]. It is worth considering the application of bias-reduction methods to these models.

### Supplementary Information

Supplementary Material 1.

## Data Availability

SAS code for the BR-GEE can be found at https://github.com/yukiotada0/brgeegor.git. Data on postoperative pain after laparoscopic cholecystectomy can be found in Lumley [14].

## Abbreviations

GEE: Generalized estimating equations
BR-GEE: Bias-reduced generalized estimating equations
MSE: Mean squared error
RMSE: Root mean squared error
