# Supplementary material for: A bias-reduced generalized estimating equation approach for proportional odds models with small-sample longitudinal ordinal data
Source: BMC Med Res Methodol. 2024 Jun 28;24:140. doi: 10.1186/s12874-024-02259-6 (PMC11212405; doi:10.1186/s12874-024-02259-6)
Supplement: Supplementary file 1 — Supplementary Material 1. [file 12874_2024_2259_MOESM1_ESM.docx]

A bias-reduced generalized estimating equation approach for proportional odds models with small-sample longitudinal ordinal data

Yukio Tada and Tosiya Sato

Additional File 1

Contents

[Appendix A. Simulation setting where number of categories is 3 and cluster size is 4. 3](#_Toc164205173)

[Appendix B. Simulation setting where number of categories is 3 and cluster size is 6. 16](#_Toc164205174)

[Appendix C. Simulation setting where number of categories is 4 and cluster size is 4. 23](#_Toc164205175)

[Appendix D. Simulation setting where number of categories is 4 and cluster size is 6. 30](#_Toc164205176)

[Appendix E. Coverage of 95% confidence interval by methods of standard error estimation. 37](#_Toc164205177)

# Appendix A. Simulation setting where number of categories is 3 and cluster size is 4.


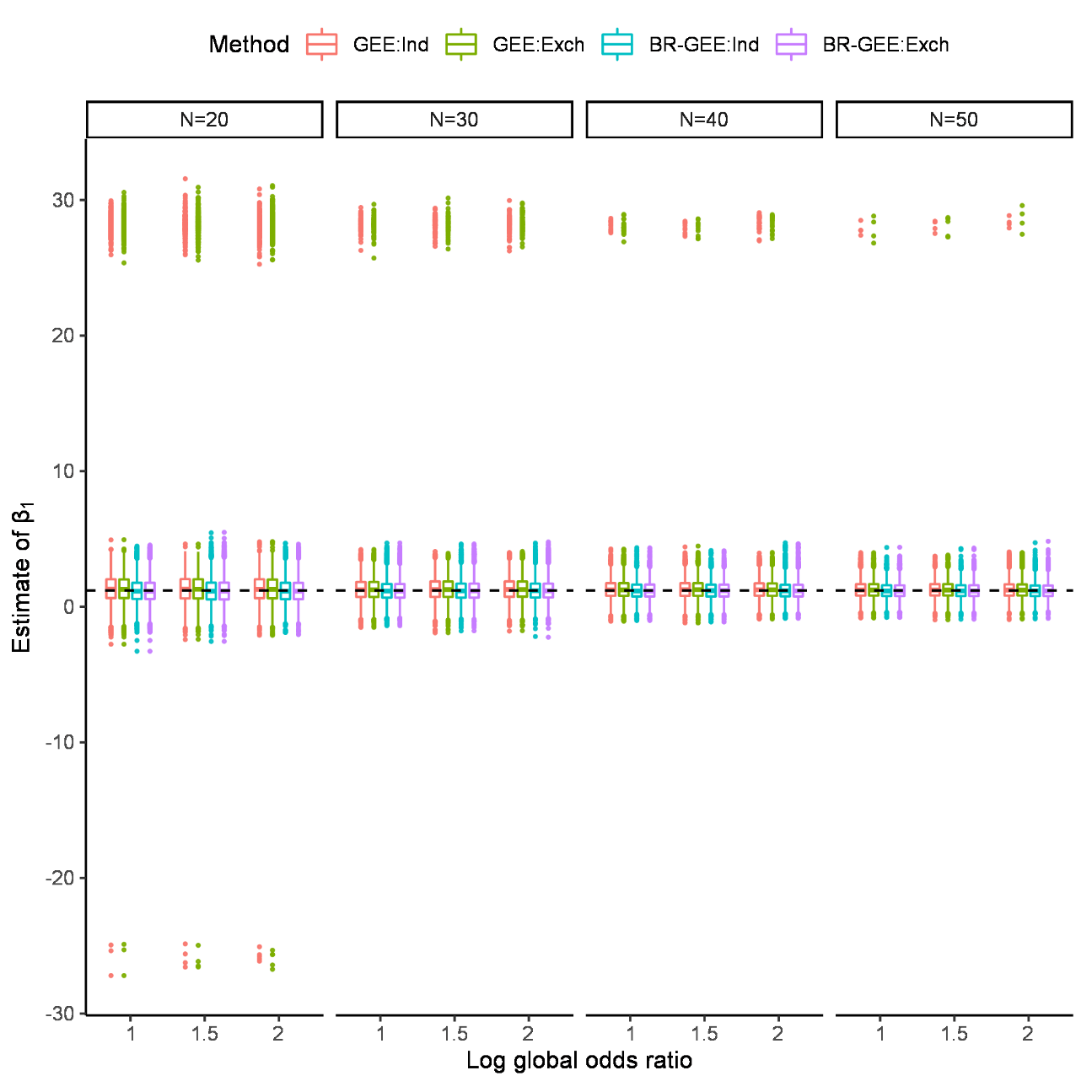


Fig. S1 Box and whisker plot for estimates of $\beta_{1}$ with true AR-type covariance structure (Scenario 1). GEE:Ind, generalized estimating equation with working independent covariance structure, GEE:Exch, generalized estimating equation with working exchangeable covariance structure, BR-GEE:Ind, bias-reduced generalized estimating equation with working independent covariance structure, BR-GEE:Exch, generalized estimating equation with working exchangeable covariance structure.


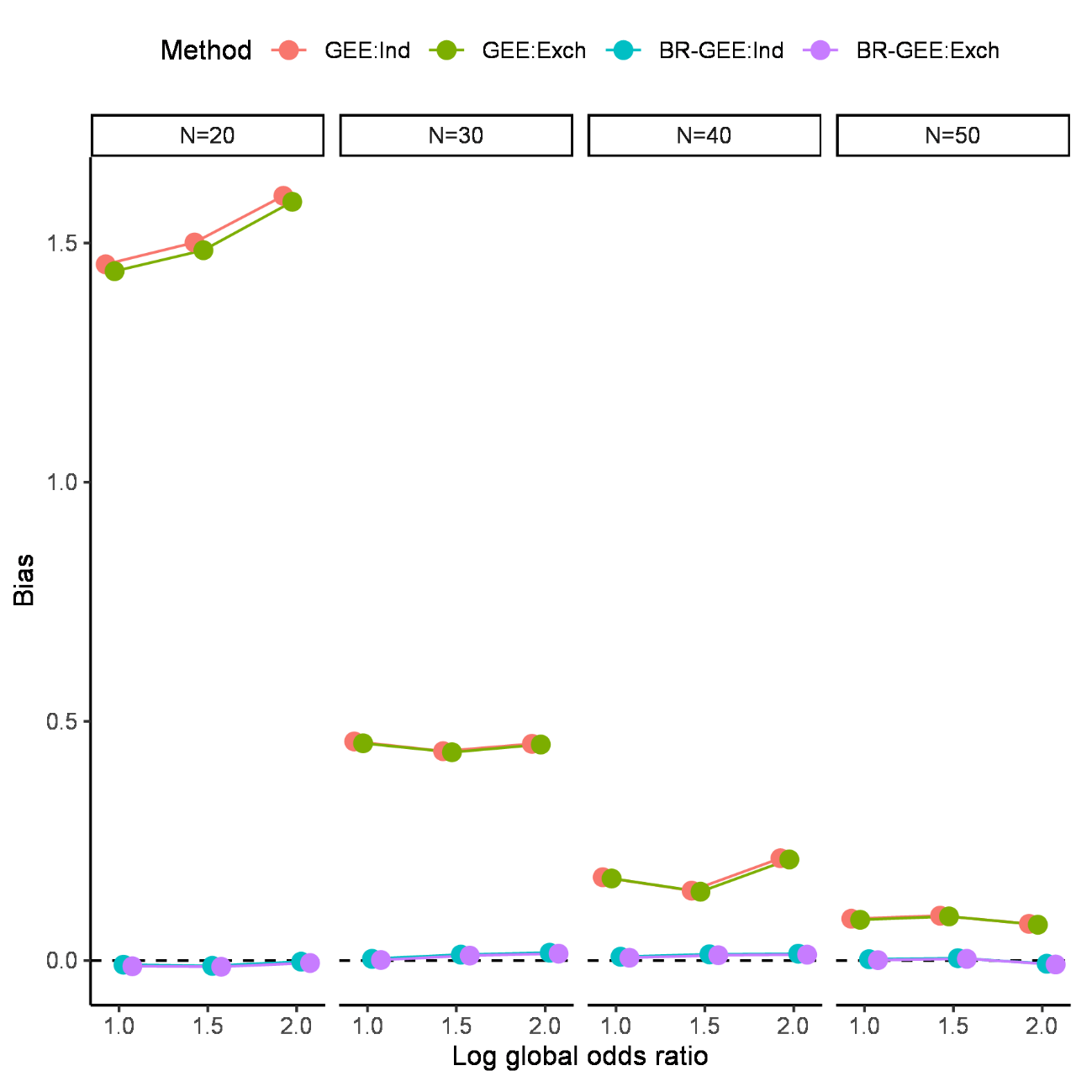


Fig. S2 Bias associated with estimates of $\beta_{1}$ with true AR-type covariance structure (Scenario 1). GEE:Ind, generalized estimating equation with working independent covariance structure, GEE:Exch, generalized estimating equation with working exchangeable covariance structure, BR-GEE:Ind, bias-reduced generalized estimating equation with working independent covariance structure, BR-GEE:Exch, generalized estimating equation with working exchangeable covariance structure.


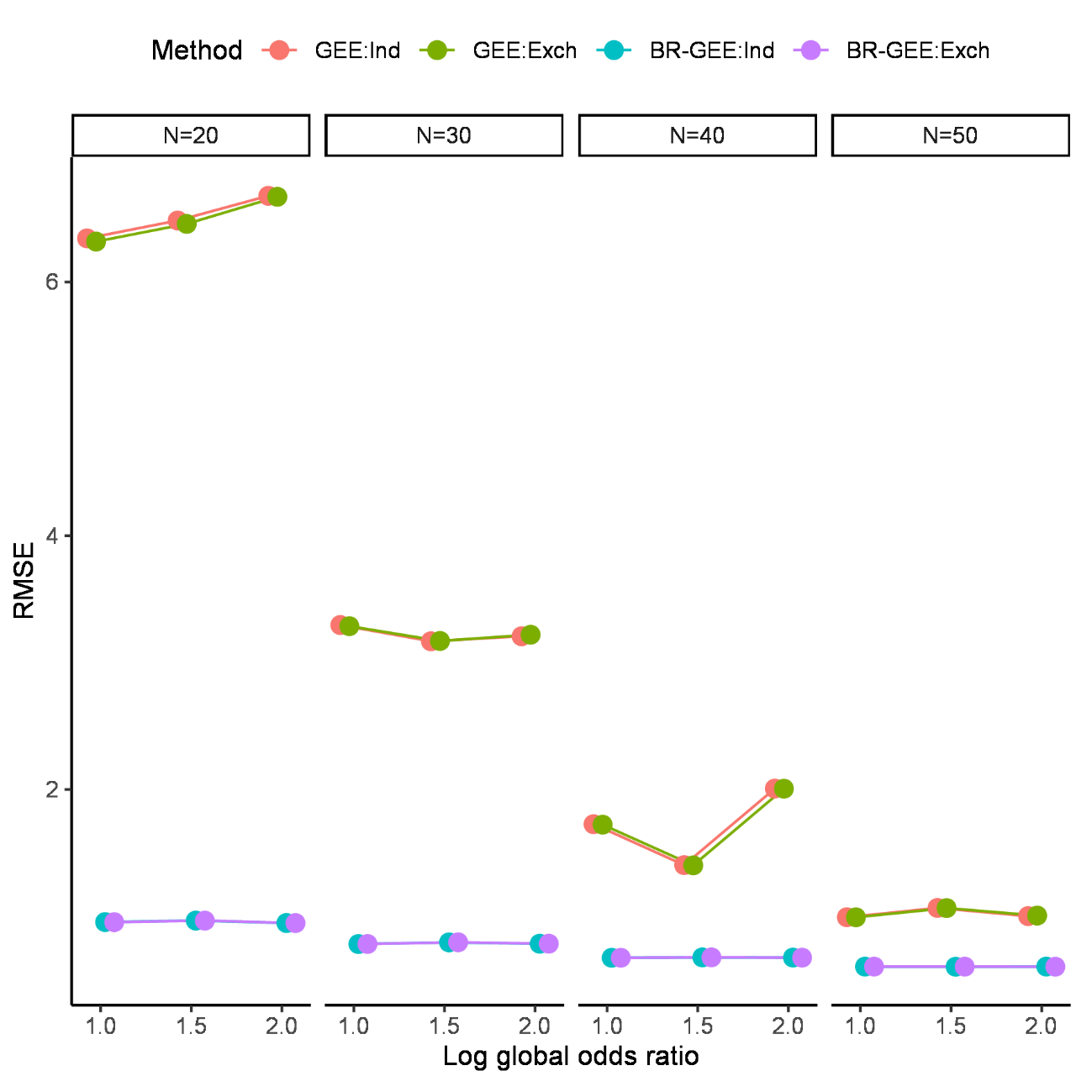


Fig. S3 RMSE associated with estimates of $\beta_{1}$ with true AR-type covariance structure (Scenario 1). GEE:Ind, generalized estimating equation with working independent covariance structure, GEE:Exch, generalized estimating equation with working exchangeable covariance structure, BR-GEE:Ind, bias-reduced generalized estimating equation with working independent covariance structure, BR-GEE:Exch, generalized estimating equation with working exchangeable covariance structure.


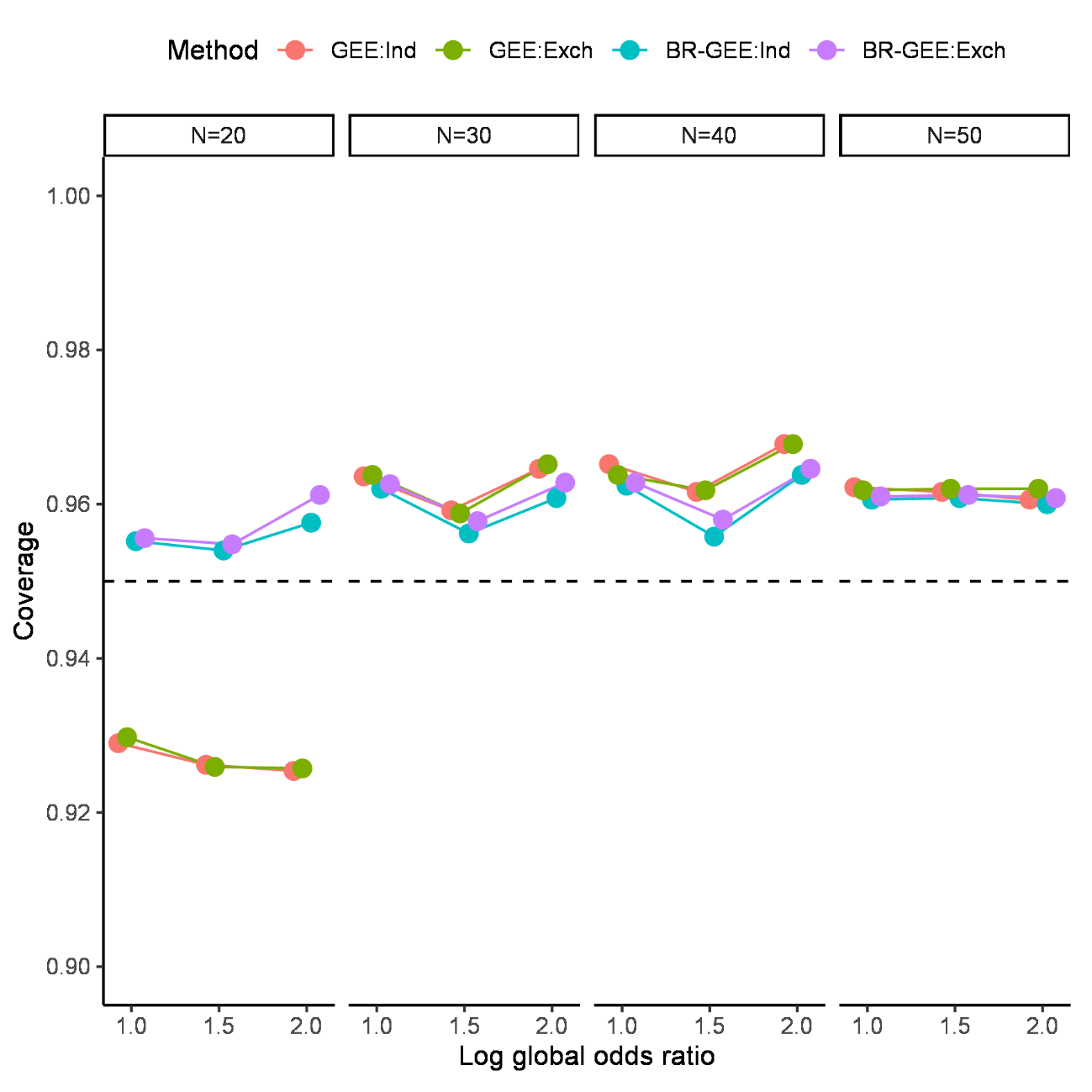


Fig. S4 95% confidence interval coverage for estimates of $\beta_{1}$ with true AR-type covariance structure (Scenario 1). GEE:Ind, generalized estimating equation with working independent covariance structure, GEE:Exch, generalized estimating equation with working exchangeable covariance structure, BR-GEE:Ind, bias-reduced generalized estimating equation with working independent covariance structure, BR-GEE:Exch, generalized estimating equation with working exchangeable covariance structure.


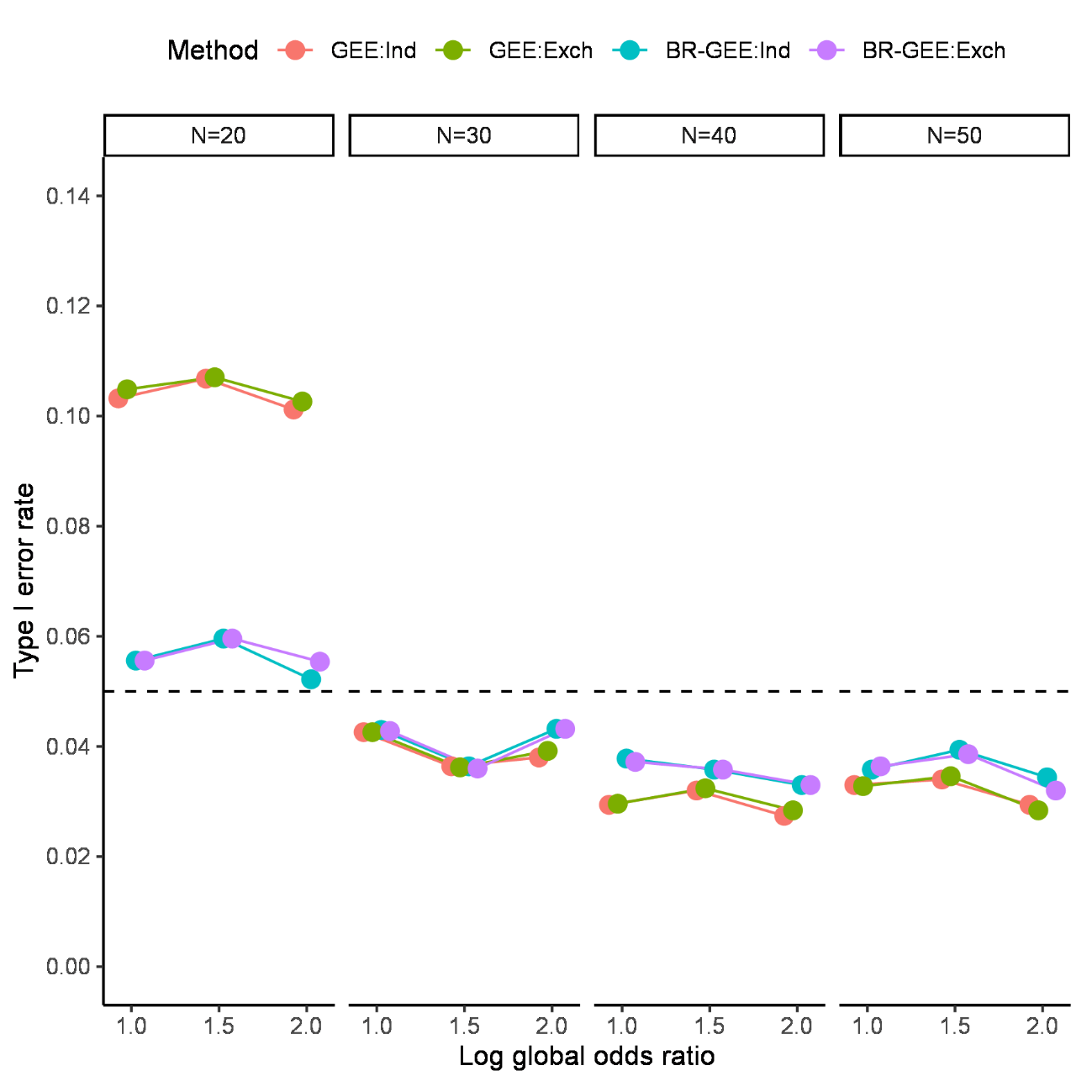


Fig. S5 Type I error of t-test of $H_{0}:\beta_{1}=0$ with true AR-type covariance structure (Scenario 2). GEE:Ind, generalized estimating equation with working independent covariance structure, GEE:Exch, generalized estimating equation with working exchangeable covariance structure, BR-GEE:Ind, bias-reduced generalized estimating equation with working independent covariance structure, BR-GEE:Exch, generalized estimating equation with working exchangeable covariance structure.


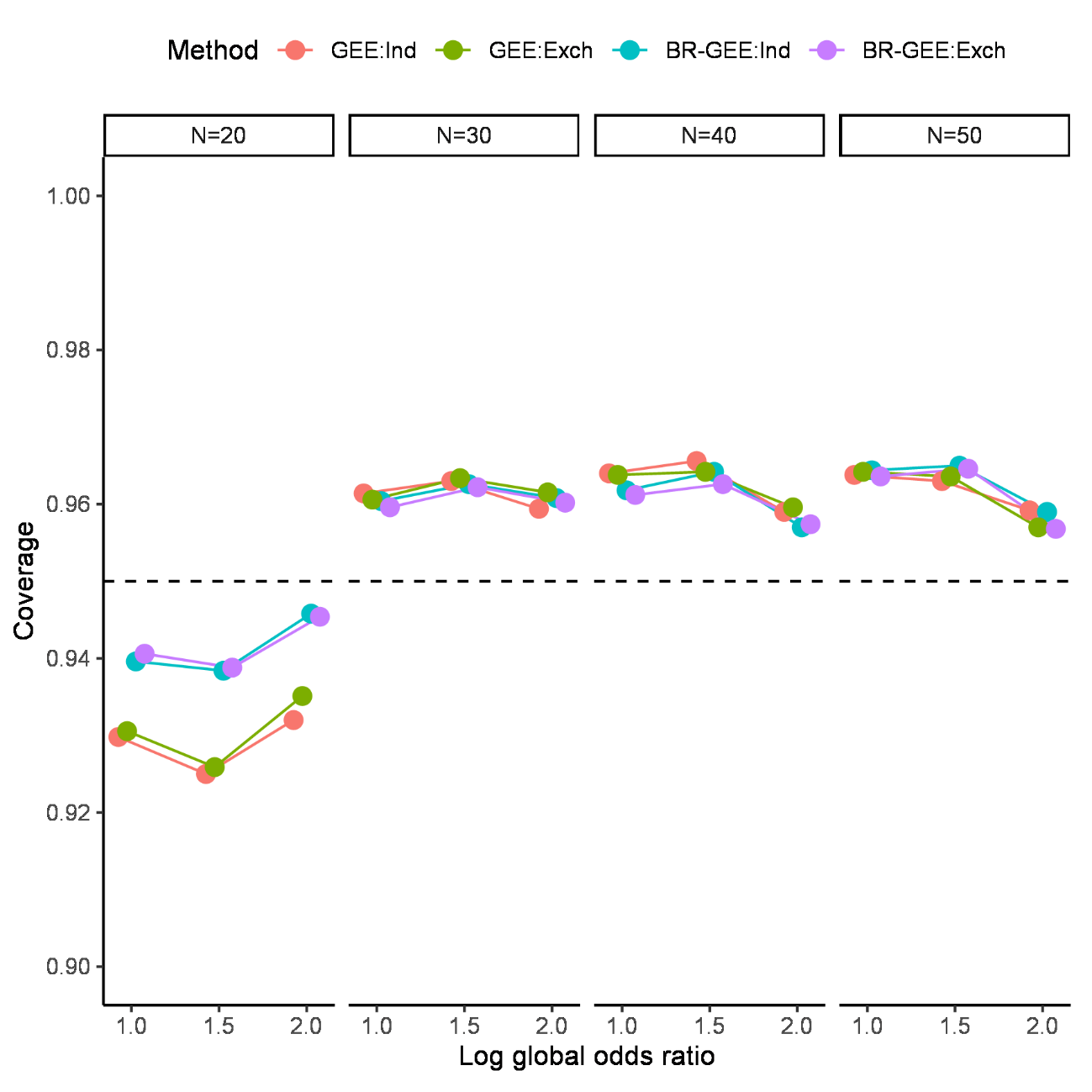


Fig. S6 95% confidence interval coverage for estimates of $\beta_{1}$ with true exchangeable covariance structure (Scenario 1, SE based on $\boldsymbol{\Sigma}_{\boldsymbol{GEE}}$). GEE:Ind, generalized estimating equation with working independent covariance structure, GEE:Exch, generalized estimating equation with working exchangeable covariance structure, BR-GEE:Ind, bias-reduced generalized estimating equation with working independent covariance structure, BR-GEE:Exch, generalized estimating equation with working exchangeable covariance structure.


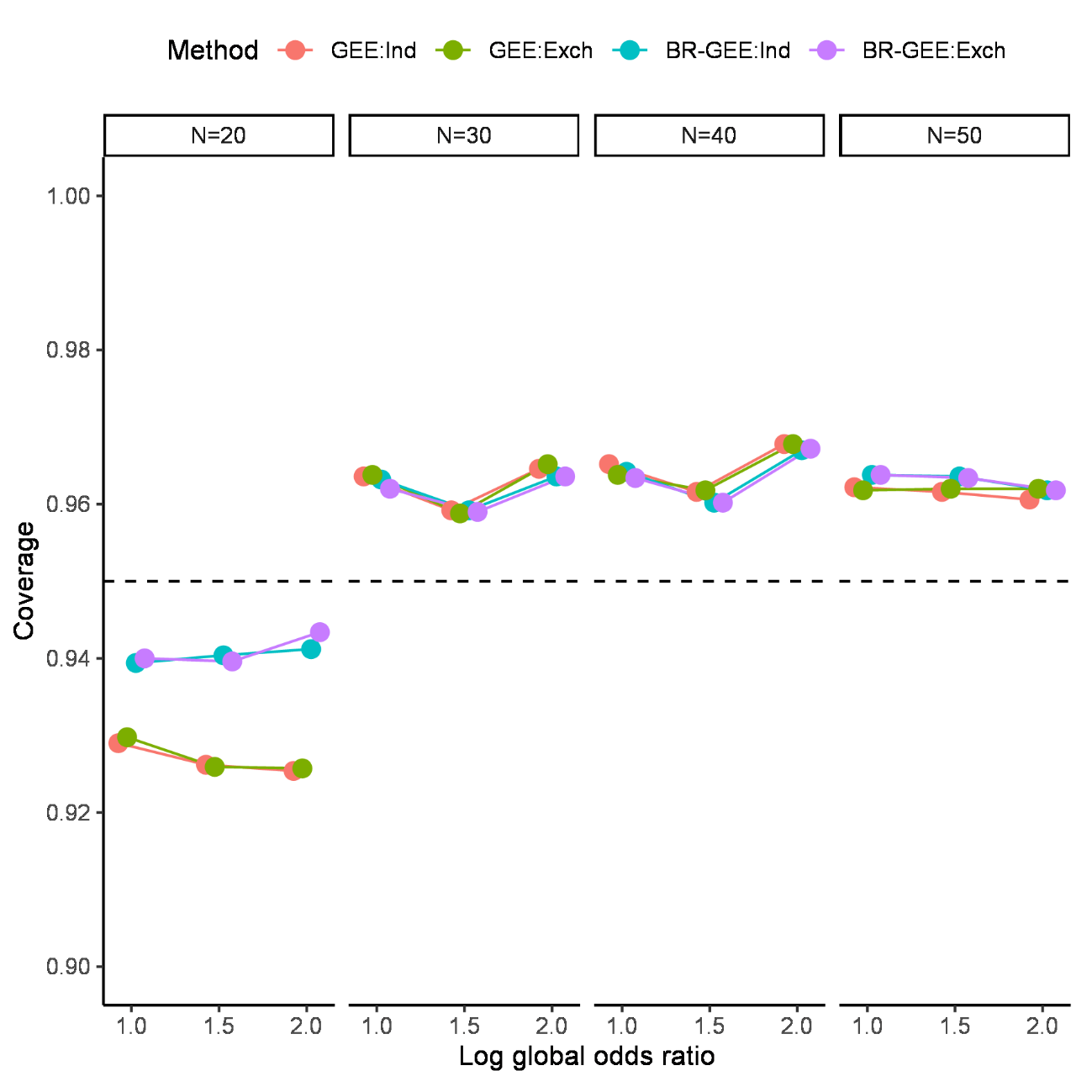


Fig. S7 95% confidence interval coverage for estimates of $\beta_{1}$ with true AR-type covariance structure (Scenario 1, SE based on $\boldsymbol{\Sigma}_{\boldsymbol{GEE}}$). GEE:Ind, generalized estimating equation with working independent covariance structure, GEE:Exch, generalized estimating equation with working exchangeable covariance structure, BR-GEE:Ind, bias-reduced generalized estimating equation with working independent covariance structure, BR-GEE:Exch, generalized estimating equation with working exchangeable covariance structure.


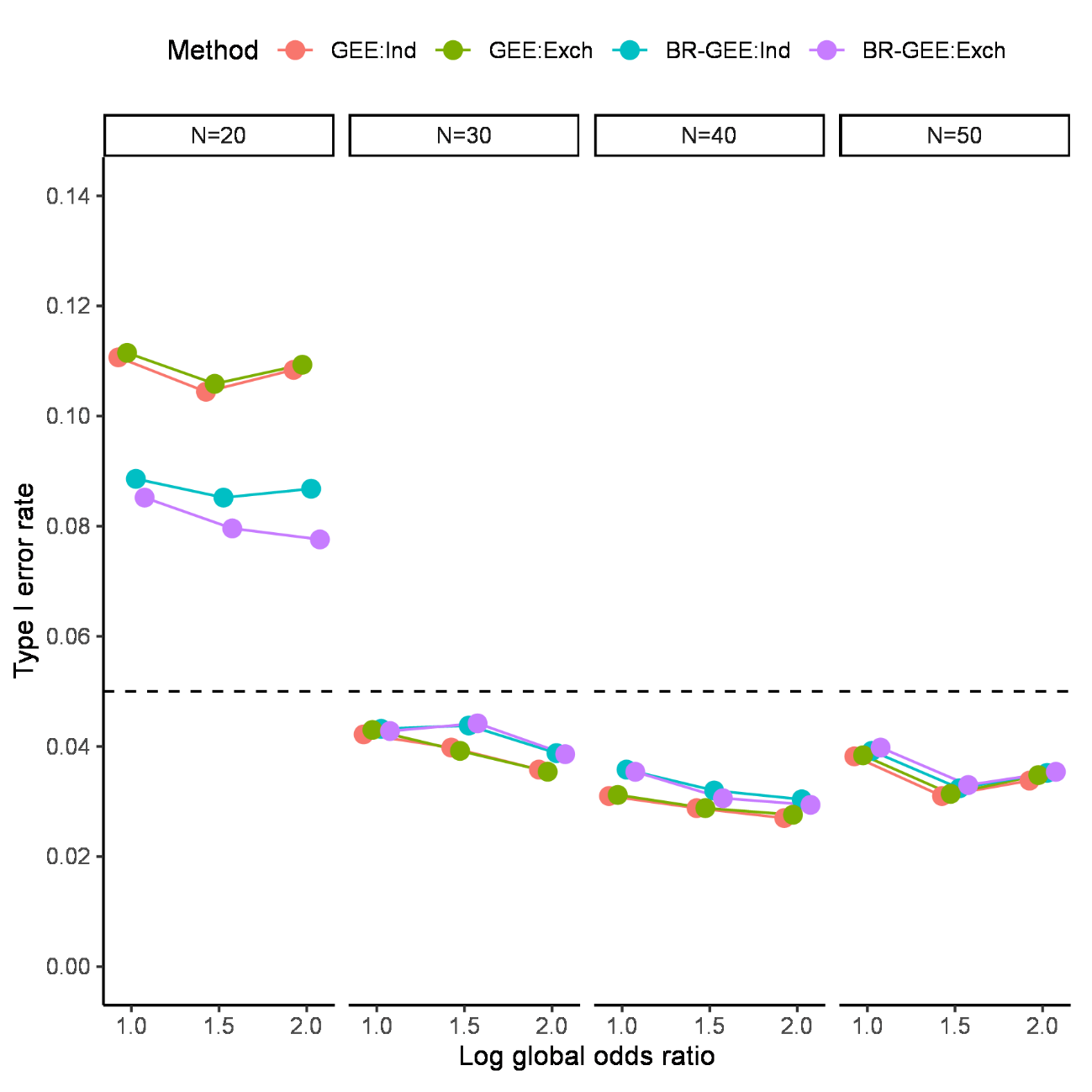


Fig. S8 Type I error of t-test of $H_{0}:\beta_{1}=0$ with true exchangeable covariance structure (Scenario 2, SE based on $\boldsymbol{\Sigma}_{\boldsymbol{GEE}}$). GEE:Ind, generalized estimating equation with working independent covariance structure, GEE:Exch, generalized estimating equation with working exchangeable covariance structure, BR-GEE:Ind, bias-reduced generalized estimating equation with working independent covariance structure, BR-GEE:Exch, generalized estimating equation with working exchangeable covariance structure.


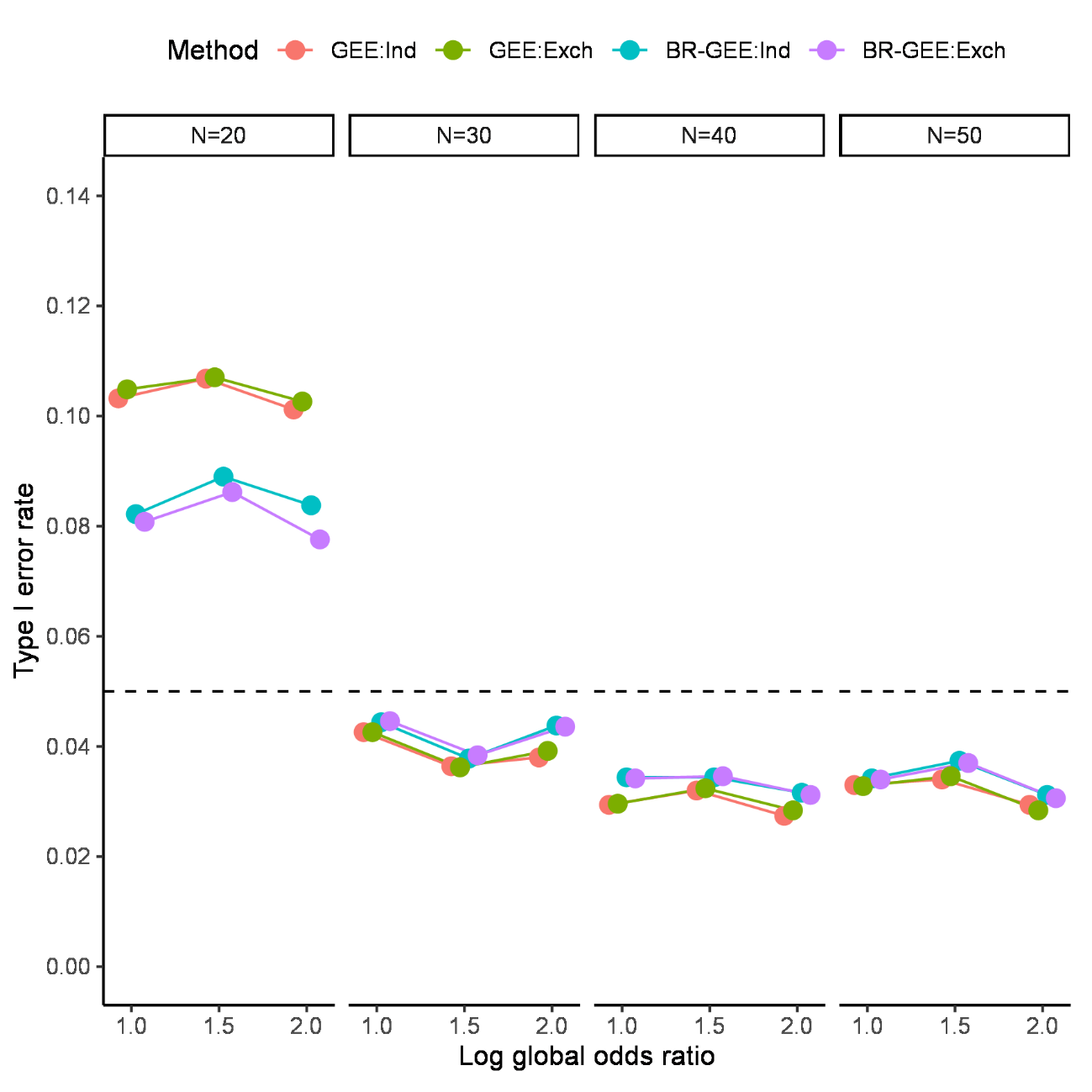


Fig. S9 Type I error of t-test of $H_{0}:\beta_{1}=0$ with true AR-type covariance structure (Scenario 2, SE based on $\boldsymbol{\Sigma}_{\boldsymbol{GEE}}$). GEE:Ind, generalized estimating equation with working independent covariance structure, GEE:Exch, generalized estimating equation with working exchangeable covariance structure, BR-GEE:Ind, bias-reduced generalized estimating equation with working independent covariance structure, BR-GEE:Exch, generalized estimating equation with working exchangeable covariance structure.

Table. S1 The percentage of convergence problem with true exchangeable covariance structure (Scenario 1)

| N | LGOR | Quasicomplete separation | GEE:Ind | GEE:Exch | BR-GEE:Ind | BR-GEE:Exch |
| --- | --- | --- | --- | --- | --- | --- |
| 20 | 1.0 | 7.84 | 7.84 | 7.84 | 0.00 | 0.00 |
|  | 1.5 | 8.06 | 8.06 | 8.06 | 0.00 | 0.00 |
|  | 2.0 | 7.30 | 7.30 | 7.30 | 0.00 | 0.00 |
| 30 | 1.0 | 1.56 | 1.56 | 1.56 | 0.00 | 0.00 |
|  | 1.5 | 1.66 | 1.66 | 1.66 | 0.00 | 0.00 |
|  | 2.0 | 1.62 | 1.62 | 1.62 | 0.00 | 0.00 |
| 40 | 1.0 | 0.30 | 0.30 | 0.30 | 0.00 | 0.00 |
|  | 1.5 | 0.28 | 0.28 | 0.28 | 0.00 | 0.00 |
|  | 2.0 | 0.40 | 0.40 | 0.40 | 0.00 | 0.00 |
| 50 | 1.0 | 0.06 | 0.06 | 0.06 | 0.00 | 0.00 |
|  | 1.5 | 0.06 | 0.06 | 0.06 | 0.00 | 0.00 |
|  | 2.0 | 0.08 | 0.08 | 0.08 | 0.00 | 0.00 |

LGOR: log global odds ratio, GEE:Ind, generalized estimating equation with working independent covariance structure, GEE:Exch, generalized estimating equation with working exchangeable covariance structure, BR-GEE:Ind, bias-reduced generalized estimating equation with working independent covariance structure, BR-GEE:Exch, generalized estimating equation with working exchangeable covariance structure.

Table. S2 The percentage of convergence problem with true exchangeable covariance structure (Scenario 2)

| N | LGOR | Quasicomplete separation | GEE:Ind | GEE:Exch | BR-GEE:Ind | BR-GEE:Exch |
| --- | --- | --- | --- | --- | --- | --- |
| 20 | 1.0 | 12.34 | 12.34 | 12.34 | 0.00 | 0.00 |
|  | 1.5 | 11.82 | 11.82 | 11.82 | 0.00 | 0.00 |
|  | 2.0 | 11.76 | 11.76 | 11.76 | 0.00 | 0.00 |
| 30 | 1.0 | 2.72 | 2.72 | 2.72 | 0.00 | 0.00 |
|  | 1.5 | 2.74 | 2.74 | 2.74 | 0.00 | 0.00 |
|  | 2.0 | 2.68 | 2.68 | 2.68 | 0.00 | 0.00 |
| 40 | 1.0 | 0.44 | 0.44 | 0.44 | 0.00 | 0.00 |
|  | 1.5 | 0.82 | 0.82 | 0.82 | 0.00 | 0.00 |
|  | 2.0 | 0.64 | 0.64 | 0.64 | 0.00 | 0.00 |
| 50 | 1.0 | 0.20 | 0.20 | 0.20 | 0.00 | 0.00 |
|  | 1.5 | 0.10 | 0.10 | 0.10 | 0.00 | 0.00 |
|  | 2.0 | 0.14 | 0.14 | 0.14 | 0.00 | 0.00 |

LGOR: log global odds ratio, GEE:Ind, generalized estimating equation with working independent covariance structure, GEE:Exch, generalized estimating equation with working exchangeable covariance structure, BR-GEE:Ind, bias-reduced generalized estimating equation with working independent covariance structure, BR-GEE:Exch, generalized estimating equation with working exchangeable covariance structure.

Table. S3 The percentage of convergence problem with true AR-type covariance structure (Scenario 1)

| N | LGOR | Quasicomplete separation | GEE:Ind | GEE:Exch | BR-GEE:Ind | BR-GEE:Exch |
| --- | --- | --- | --- | --- | --- | --- |
| 20 | 1.0 | 7.68 | 7.68 | 7.68 | 0.00 | 0.00 |
|  | 1.5 | 8.10 | 8.10 | 8.10 | 0.00 | 0.00 |
|  | 2.0 | 7.98 | 7.98 | 7.98 | 0.00 | 0.00 |
| 30 | 1.0 | 1.72 | 1.72 | 1.72 | 0.00 | 0.00 |
|  | 1.5 | 1.72 | 1.72 | 1.72 | 0.00 | 0.00 |
|  | 2.0 | 1.52 | 1.52 | 1.52 | 0.00 | 0.00 |
| 40 | 1.0 | 0.42 | 0.42 | 0.42 | 0.00 | 0.00 |
|  | 1.5 | 0.24 | 0.24 | 0.24 | 0.00 | 0.00 |
|  | 2.0 | 0.52 | 0.52 | 0.52 | 0.00 | 0.00 |
| 50 | 1.0 | 0.08 | 0.08 | 0.08 | 0.00 | 0.00 |
|  | 1.5 | 0.10 | 0.10 | 0.10 | 0.00 | 0.00 |
|  | 2.0 | 0.08 | 0.08 | 0.08 | 0.00 | 0.00 |

LGOR: log global odds ratio, GEE:Ind, generalized estimating equation with working independent covariance structure, GEE:Exch, generalized estimating equation with working exchangeable covariance structure, BR-GEE:Ind, bias-reduced generalized estimating equation with working independent covariance structure, BR-GEE:Exch, generalized estimating equation with working exchangeable covariance structure.

Table. S4 The percentage of convergence problem with true AR-type covariance structure (Scenario 2)

| N | LGOR | Quasicomplete separation | GEE:Ind | GEE:Exch | BR-GEE:Ind | BR-GEE:Exch |
| --- | --- | --- | --- | --- | --- | --- |
| 20 | 1.0 | 11.50 | 11.50 | 11.50 | 0.00 | 0.00 |
|  | 1.5 | 11.66 | 11.66 | 11.66 | 0.00 | 0.00 |
|  | 2.0 | 10.80 | 10.80 | 10.80 | 0.00 | 0.00 |
| 30 | 1.0 | 2.72 | 2.72 | 2.72 | 0.00 | 0.00 |
|  | 1.5 | 2.38 | 2.38 | 2.38 | 0.00 | 0.00 |
|  | 2.0 | 2.48 | 2.48 | 2.48 | 0.00 | 0.00 |
| 40 | 1.0 | 0.72 | 0.72 | 0.72 | 0.00 | 0.00 |
|  | 1.5 | 0.62 | 0.62 | 0.62 | 0.00 | 0.00 |
|  | 2.0 | 0.56 | 0.56 | 0.56 | 0.00 | 0.00 |
| 50 | 1.0 | 0.10 | 0.10 | 0.10 | 0.00 | 0.00 |
|  | 1.5 | 0.16 | 0.16 | 0.16 | 0.00 | 0.00 |
|  | 2.0 | 0.12 | 0.12 | 0.12 | 0.00 | 0.00 |

LGOR: log global odds ratio, GEE:Ind, generalized estimating equation with working independent covariance structure, GEE:Exch, generalized estimating equation with working exchangeable covariance structure, BR-GEE:Ind, bias-reduced generalized estimating equation with working independent covariance structure, BR-GEE:Exch, generalized estimating equation with working exchangeable covariance structure.

# Appendix B. Simulation setting where number of categories is 3 and cluster size is 6.

Under true regression model presented below (Scenario 3, K=3 and n=6), the simulation results assuming number of categories 3, cluster size 6 and sample size N=20 or 50 are shown. For this set of simulations, the expected marginal probabilities at the last time point are (0.75, 0.15, 0.10). Data generation was repeated 1000 times.

$\begin{matrix} \mathrm{logit}\left( \gamma_{itk} \right)=\beta_{0k}+\beta_{1}\mathrm{Trt}_{i}+\sum_{s=1}^{5} \left\{ \beta_{s+1}I\left( \mathrm{Time}_{it}=s \right)+\beta_{s+6}\mathrm{Trt}_{i}\times I\left( \mathrm{Time}_{it}=s \right) \right\} & (k=1,2) \end{matrix}$ with $\left( \beta_{01},\beta_{02},\beta_{1},\ldots,\beta_{11} \right)=\left( -0.1, 1, 1.2, -0.9, -0.6, -0.3, -0.2, -0.1, -0.3, -0.2, -0.2, -0.1, -0.1 \right)$.


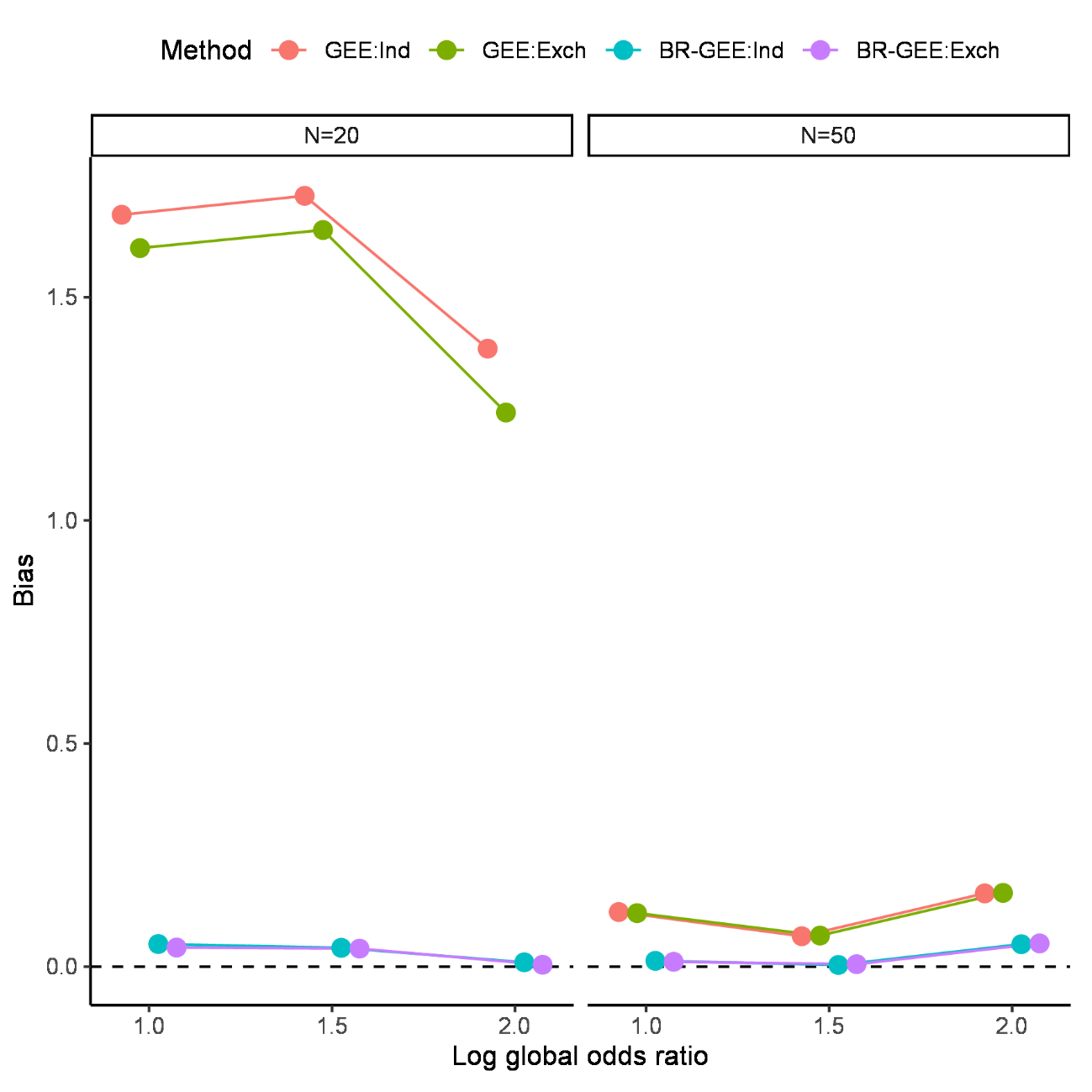


Fig. S10 Bias associated with estimates of $\beta_{1}$ with true exchangeable covariance structure (Scenario 3, K=3 and n=6). GEE:Ind, generalized estimating equation with working independent covariance structure, GEE:Exch, generalized estimating equation with working exchangeable covariance structure, BR-GEE:Ind, bias-reduced generalized estimating equation with working independent covariance structure, BR-GEE:Exch, generalized estimating equation with working exchangeable covariance structure.


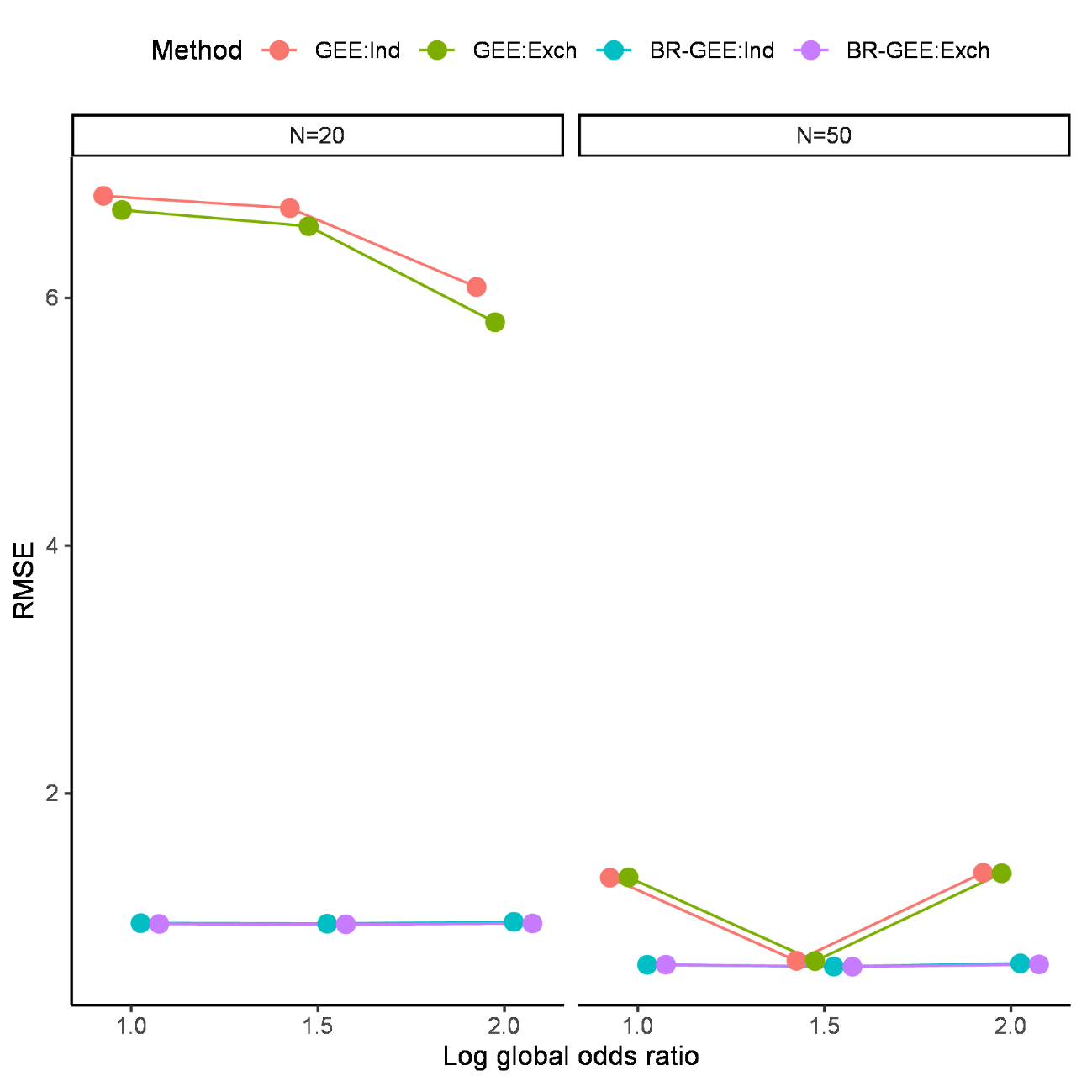


Fig. S11 RMSE associated with estimates of $\beta_{1}$ with true exchangeable covariance structure (Scenario 3, K=3 and n=6). GEE:Ind, generalized estimating equation with working independent covariance structure, GEE:Exch, generalized estimating equation with working exchangeable covariance structure, BR-GEE:Ind, bias-reduced generalized estimating equation with working independent covariance structure, BR-GEE:Exch, generalized estimating equation with working exchangeable covariance structure.


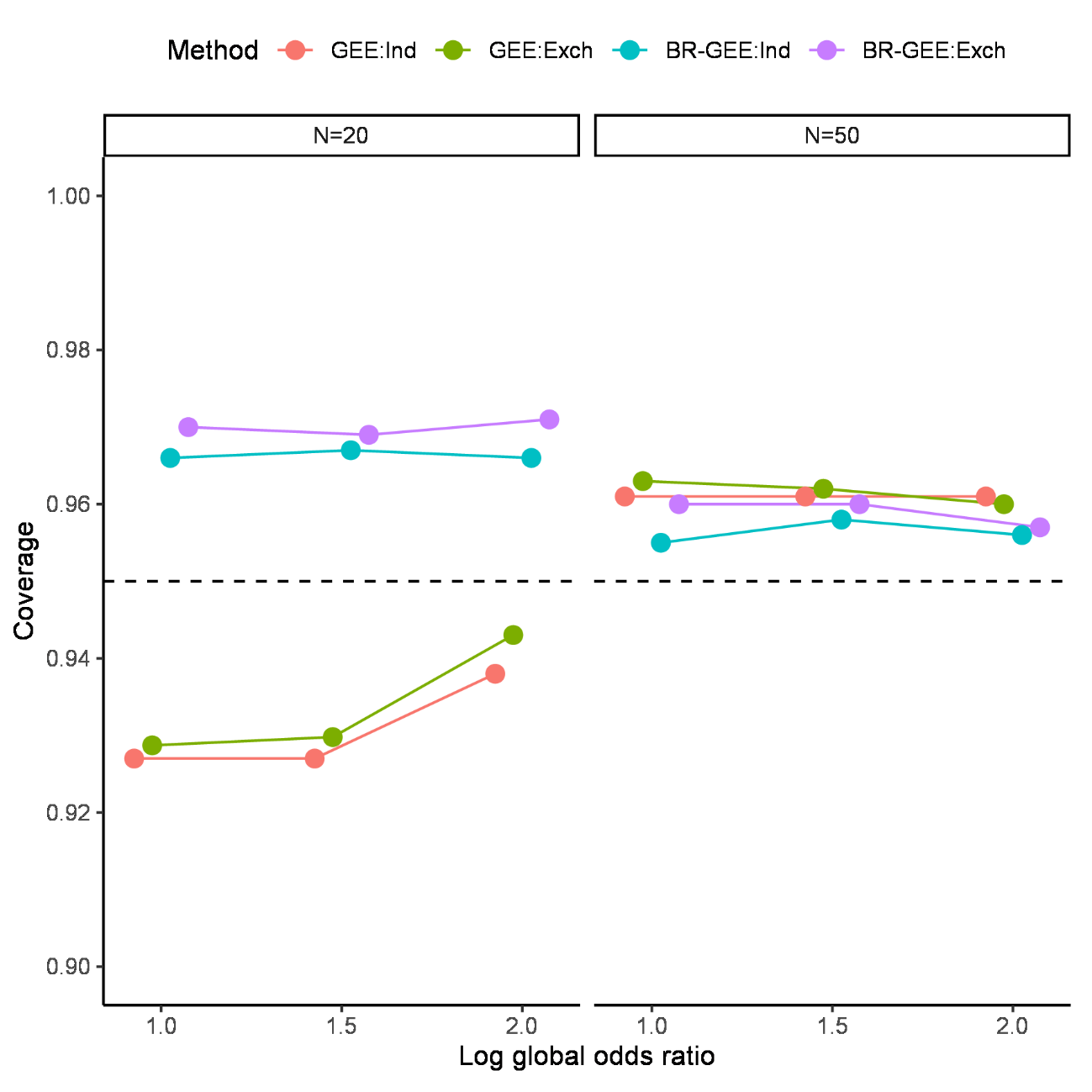


Fig. S12 95% confidence interval coverage for estimates of $\beta_{1}$ with true exchangeable covariance structure (Scenario 3, K=3 and n=6). GEE:Ind, generalized estimating equation with working independent covariance structure, GEE:Exch, generalized estimating equation with working exchangeable covariance structure, BR-GEE:Ind, bias-reduced generalized estimating equation with working independent covariance structure, BR-GEE:Exch, generalized estimating equation with working exchangeable covariance structure.


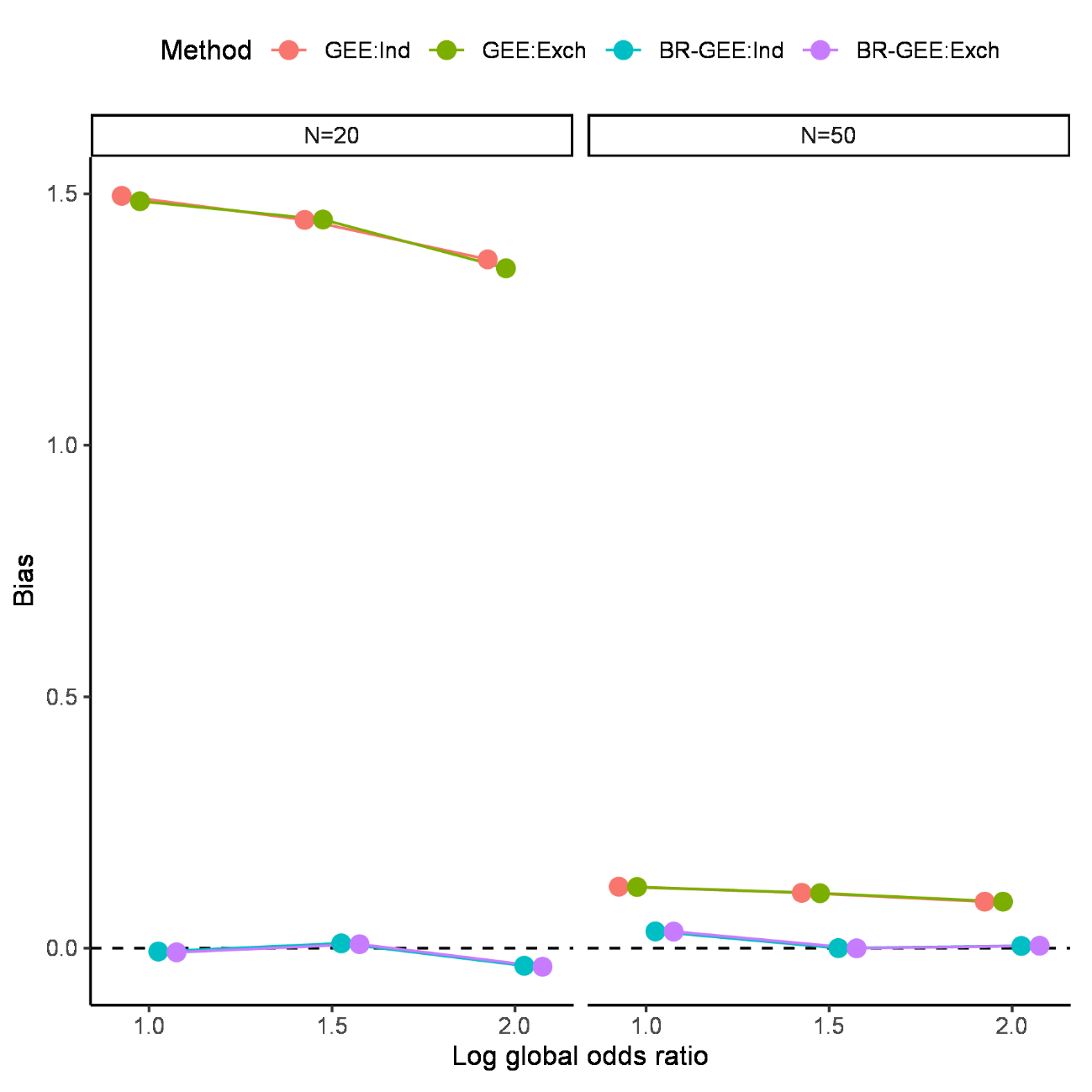


Fig. S13 Bias associated with estimates of $\beta_{1}$ with true AR-type covariance structure (Scenario 3, K=3 and n=6). GEE:Ind, generalized estimating equation with working independent covariance structure, GEE:Exch, generalized estimating equation with working exchangeable covariance structure, BR-GEE:Ind, bias-reduced generalized estimating equation with working independent covariance structure, BR-GEE:Exch, generalized estimating equation with working exchangeable covariance structure.


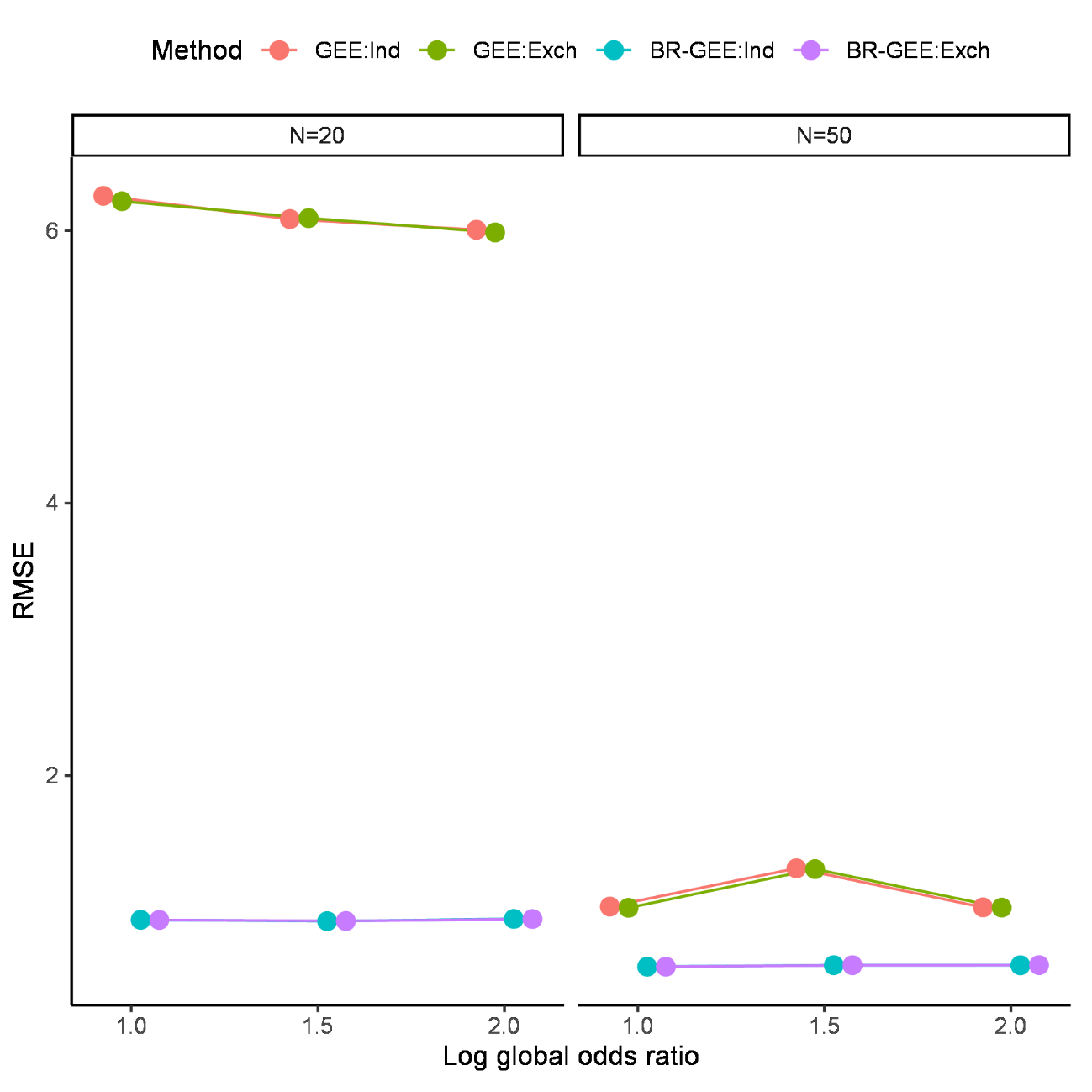


Fig. S14 RMSE associated with estimates of $\beta_{1}$ with true AR-type covariance structure (Scenario 3, K=3 and n=6). GEE:Ind, generalized estimating equation with working independent covariance structure, GEE:Exch, generalized estimating equation with working exchangeable covariance structure, BR-GEE:Ind, bias-reduced generalized estimating equation with working independent covariance structure, BR-GEE:Exch, generalized estimating equation with working exchangeable covariance structure.


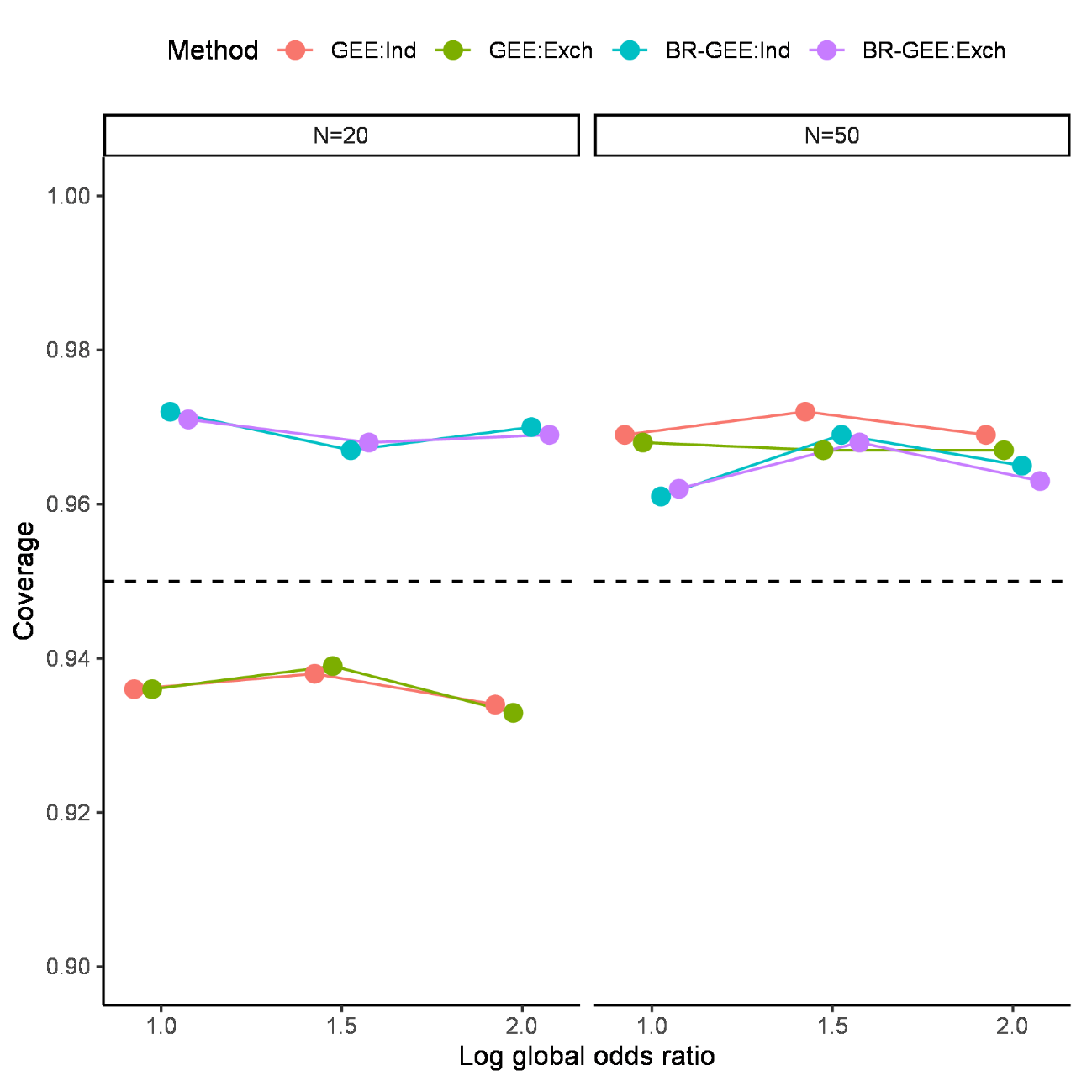


Fig. S15 95% confidence interval coverage for estimates of $\beta_{1}$ with true AR-type covariance structure (Scenario 3, K=3 and n=6). GEE:Ind, generalized estimating equation with working independent covariance structure, GEE:Exch, generalized estimating equation with working exchangeable covariance structure, BR-GEE:Ind, bias-reduced generalized estimating equation with working independent covariance structure, BR-GEE:Exch, generalized estimating equation with working exchangeable covariance structure.

# Appendix C. Simulation setting where number of categories is 4 and cluster size is 4.

Under true regression model presented below (Scenario 4, K=4 and n=4), the simulation results assuming number of categories 4, cluster size 4 and sample size N=20 or 50 are shown. For this set of simulations, the expected marginal probabilities at the last time point are (0.65, 0.14, 0.11, 0.10). Data generation was repeated 1000 times.

$\begin{matrix} \mathrm{logit}\left( \gamma_{itk} \right)=\beta_{0k}+\beta_{1}\mathrm{Trt}_{i}+\sum_{s=1}^{3} \left\{ \beta_{s+1}I\left( \mathrm{Time}_{it}=s \right)+\beta_{s+4}\mathrm{Trt}_{i}\times I\left( \mathrm{Time}_{it}=s \right) \right\} & (k=1,2,3) \end{matrix}$ with $\left( \beta_{01},\beta_{02},\beta_{03},\beta_{1},\ldots,\beta_{7} \right)=\left( -0.6, 0.1,1, 1.2, -0.9, -0.6, -0.3, -0.3, -0.2, -0.1 \right)$.


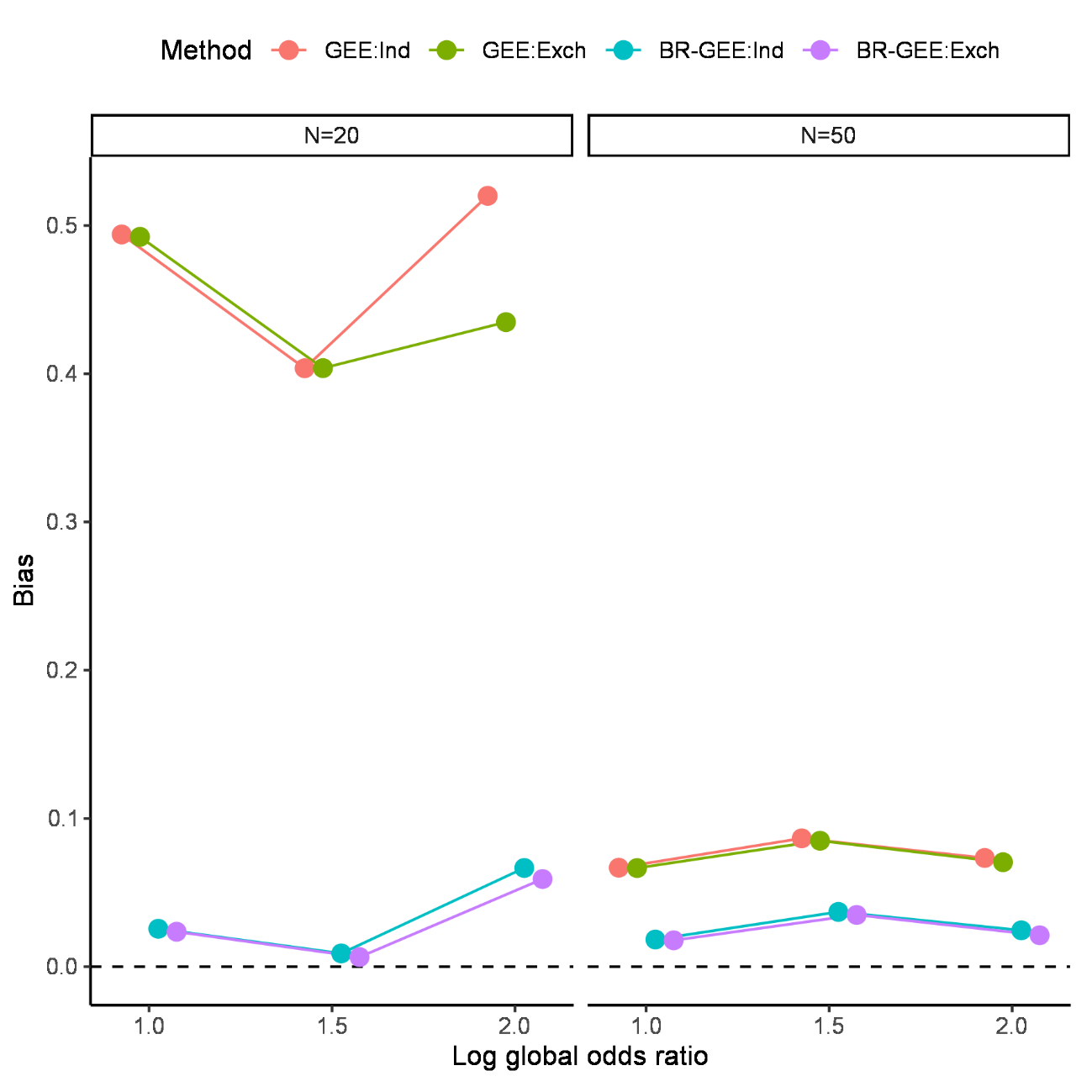


Fig. S16 Bias associated with estimates of $\beta_{1}$ with true exchangeable covariance structure (Scenario 4, K=4 and n=4). GEE:Ind, generalized estimating equation with working independent covariance structure, GEE:Exch, generalized estimating equation with working exchangeable covariance structure, BR-GEE:Ind, bias-reduced generalized estimating equation with working independent covariance structure, BR-GEE:Exch, generalized estimating equation with working exchangeable covariance structure.


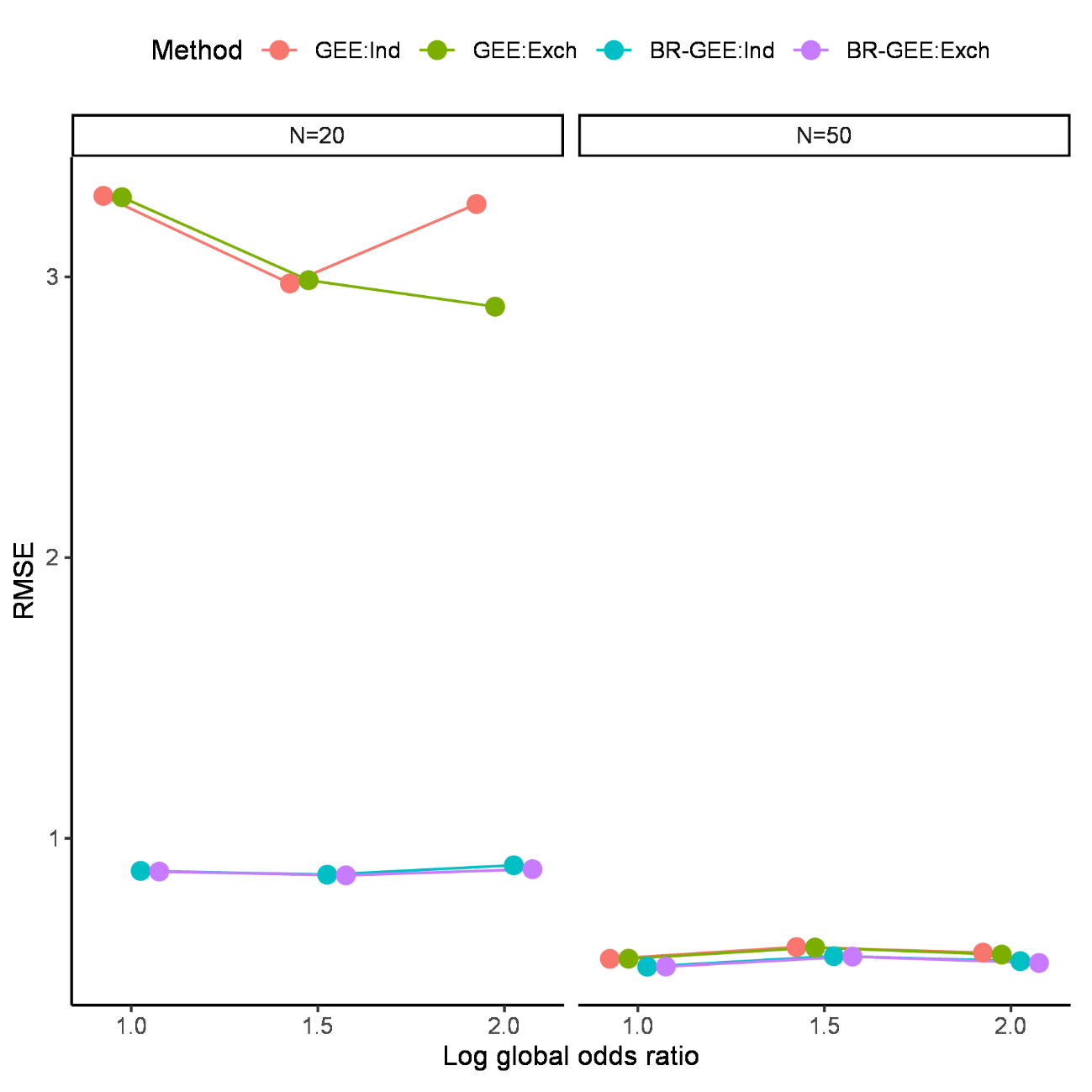


Fig. S17 RMSE associated with estimates of $\beta_{1}$ with true exchangeable covariance structure (Scenario 4, K=4 and n=4). GEE:Ind, generalized estimating equation with working independent covariance structure, GEE:Exch, generalized estimating equation with working exchangeable covariance structure, BR-GEE:Ind, bias-reduced generalized estimating equation with working independent covariance structure, BR-GEE:Exch, generalized estimating equation with working exchangeable covariance structure.


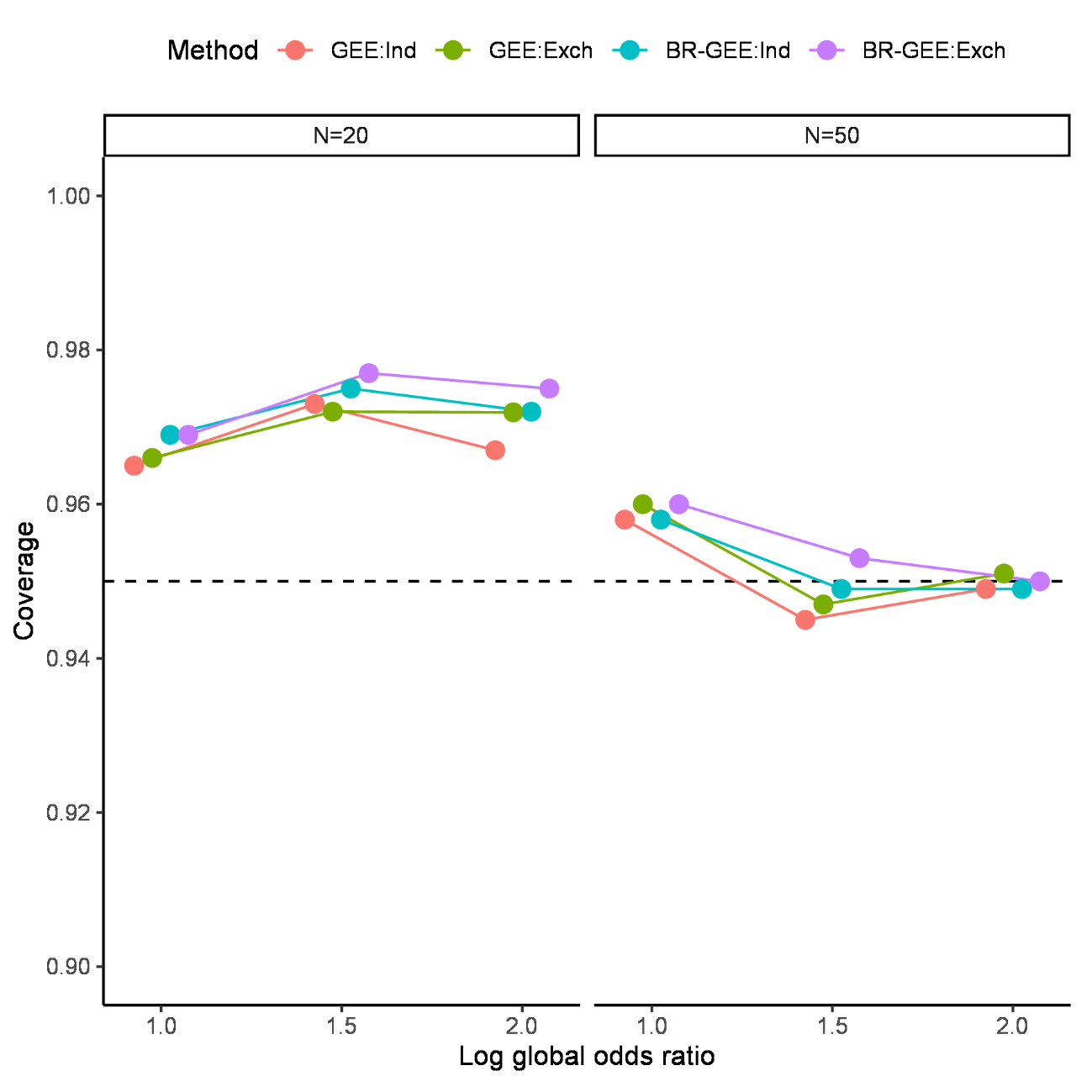


Fig. S18 95% confidence interval coverage for estimates of $\beta_{1}$ with true exchangeable covariance structure (Scenario 4, K=4 and n=4). GEE:Ind, generalized estimating equation with working independent covariance structure, GEE:Exch, generalized estimating equation with working exchangeable covariance structure, BR-GEE:Ind, bias-reduced generalized estimating equation with working independent covariance structure, BR-GEE:Exch, generalized estimating equation with working exchangeable covariance structure.


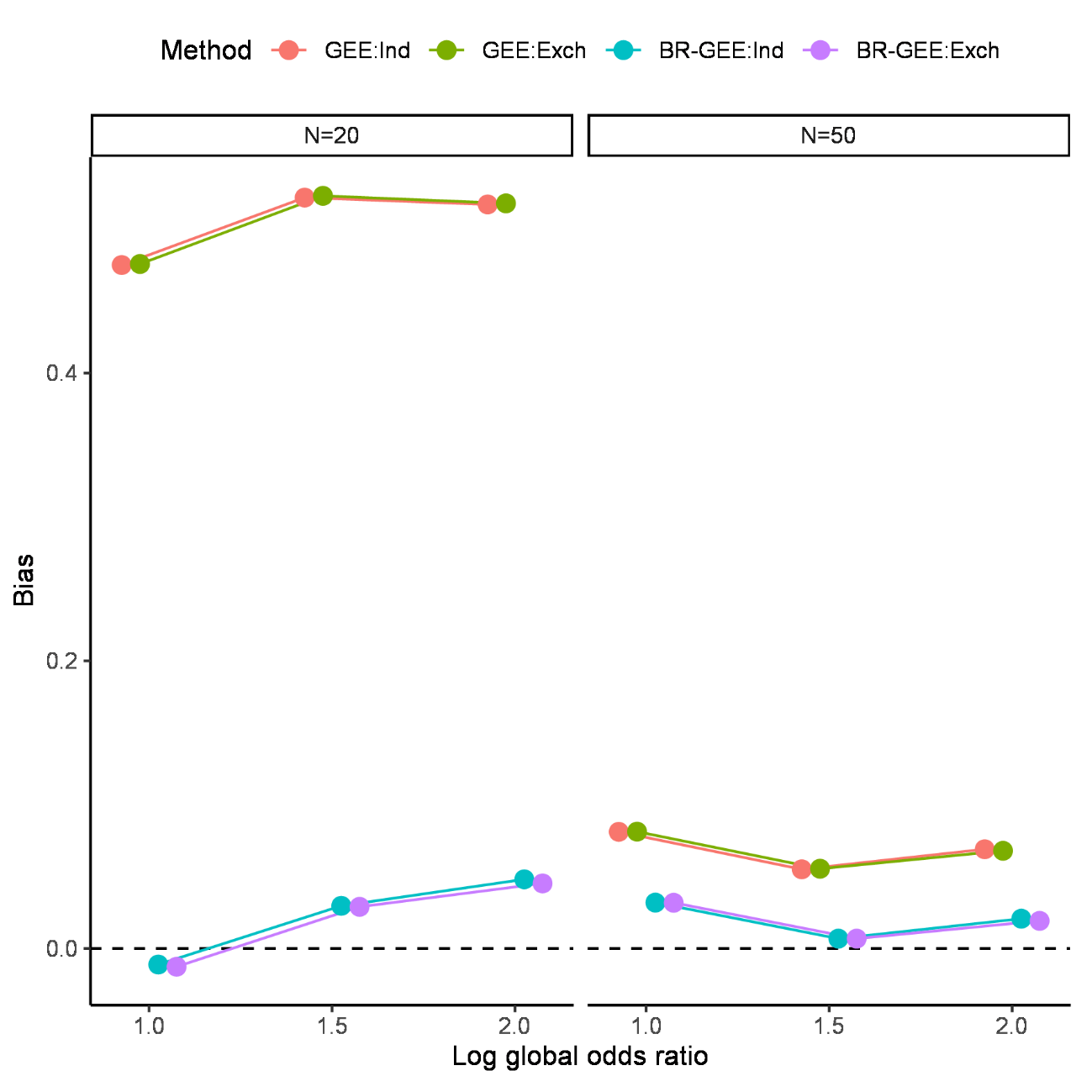


Fig. S19 Bias associated with estimates of $\beta_{1}$ with true AR-type covariance structure (Scenario 4, K=4 and n=4). GEE:Ind, generalized estimating equation with working independent covariance structure, GEE:Exch, generalized estimating equation with working exchangeable covariance structure, BR-GEE:Ind, bias-reduced generalized estimating equation with working independent covariance structure, BR-GEE:Exch, generalized estimating equation with working exchangeable covariance structure.


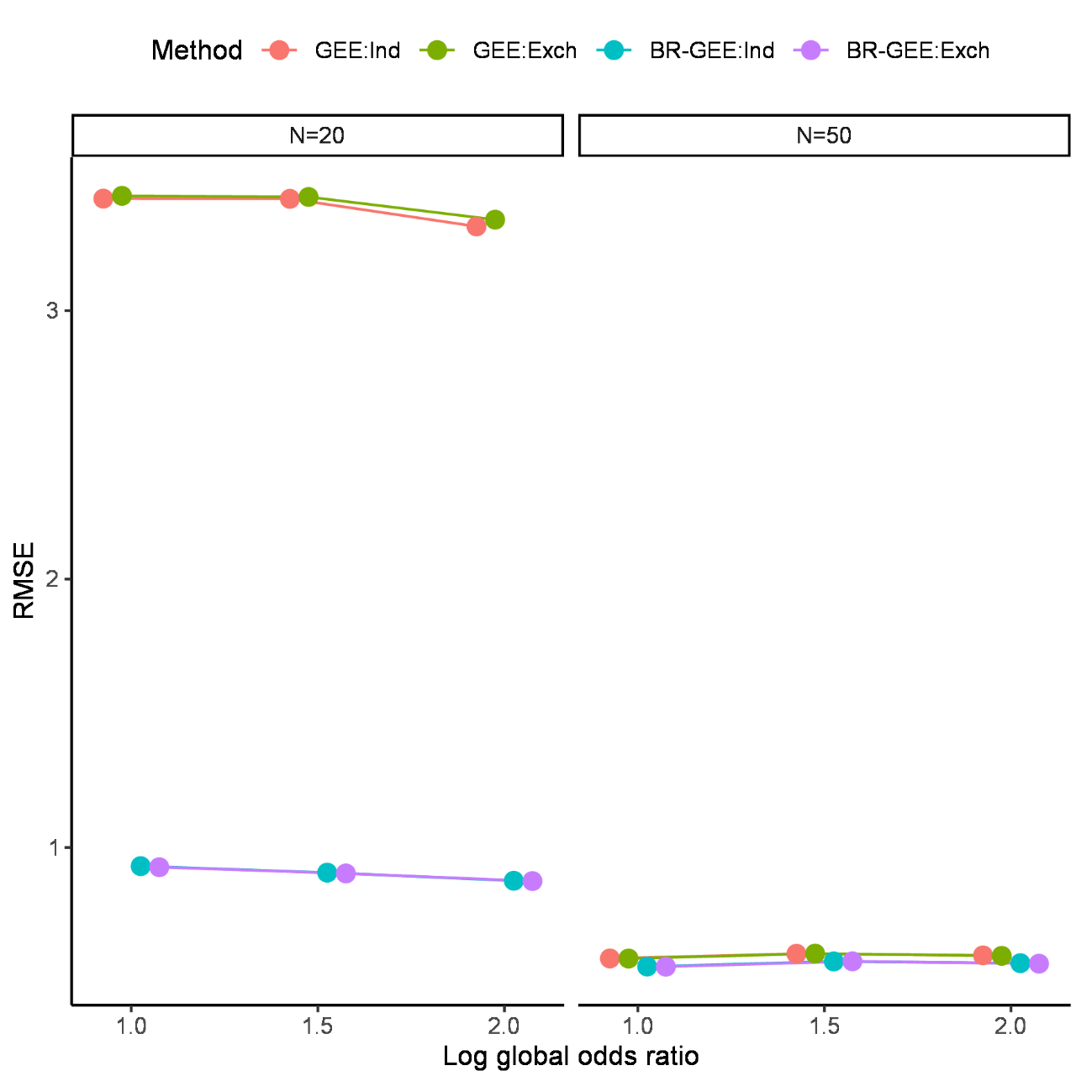


Fig. S20 RMSE associated with estimates of $\beta_{1}$ with true AR-type covariance structure (Scenario 4, K=4 and n=4). GEE:Ind, generalized estimating equation with working independent covariance structure, GEE:Exch, generalized estimating equation with working exchangeable covariance structure, BR-GEE:Ind, bias-reduced generalized estimating equation with working independent covariance structure, BR-GEE:Exch, generalized estimating equation with working exchangeable covariance structure.


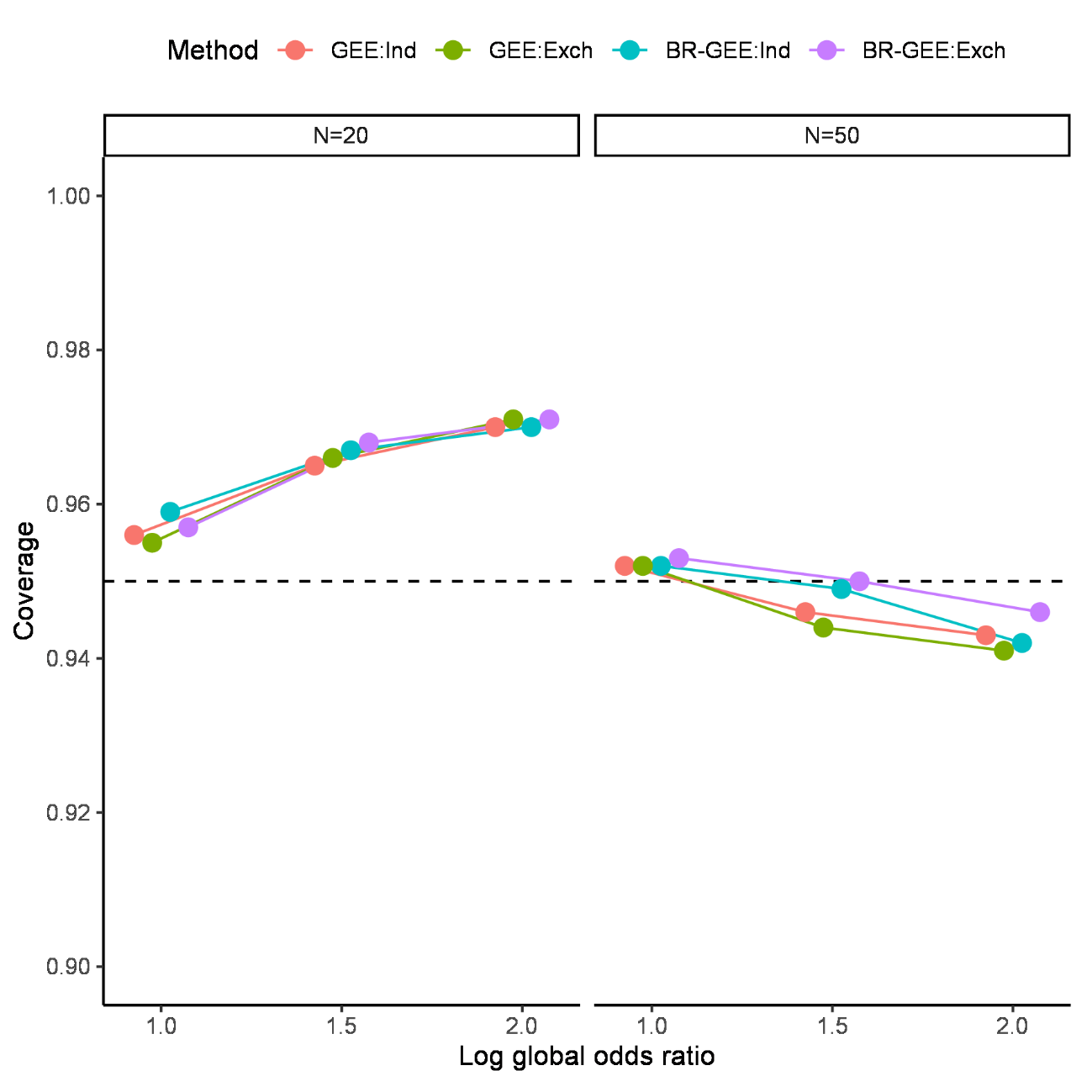


Fig. S21 95% confidence interval coverage for estimates of $\beta_{1}$ with true AR-type covariance structure (Scenario 4, K=4 and n=4). GEE:Ind, generalized estimating equation with working independent covariance structure, GEE:Exch, generalized estimating equation with working exchangeable covariance structure, BR-GEE:Ind, bias-reduced generalized estimating equation with working independent covariance structure, BR-GEE:Exch, generalized estimating equation with working exchangeable covariance structure.

# Appendix D. Simulation setting where number of categories is 4 and cluster size is 6.

Under true regression model presented below (Scenario 5, K=4 and n=6), the simulation results assuming number of categories 4, cluster size 6 and sample size N=20 or 50 are shown. For this set of simulations, the expected marginal probabilities at the last time point are (0.65, 0.14, 0.11, 0.10). Data generation was repeated 1000 times.

$\begin{matrix} \mathrm{logit}\left( \gamma_{itk} \right)=\beta_{0k}+\beta_{1}\mathrm{Trt}_{i}+\sum_{s=1}^{5} \left\{ \beta_{s+1}I\left( \mathrm{Time}_{it}=s \right)+\beta_{s+6}\mathrm{Trt}_{i}\times I\left( \mathrm{Time}_{it}=s \right) \right\} & (k=1,2,3) \end{matrix}$ with $\left( \beta_{01},\beta_{02},\beta_{03},\beta_{1},\ldots,\beta_{11} \right)=\left( -0.6,0.1, 1, 1.2, -0.9, -0.6, -0.3, -0.2, -0.1, -0.3, -0.2, -0.2, -0.1, -0.1 \right)$.


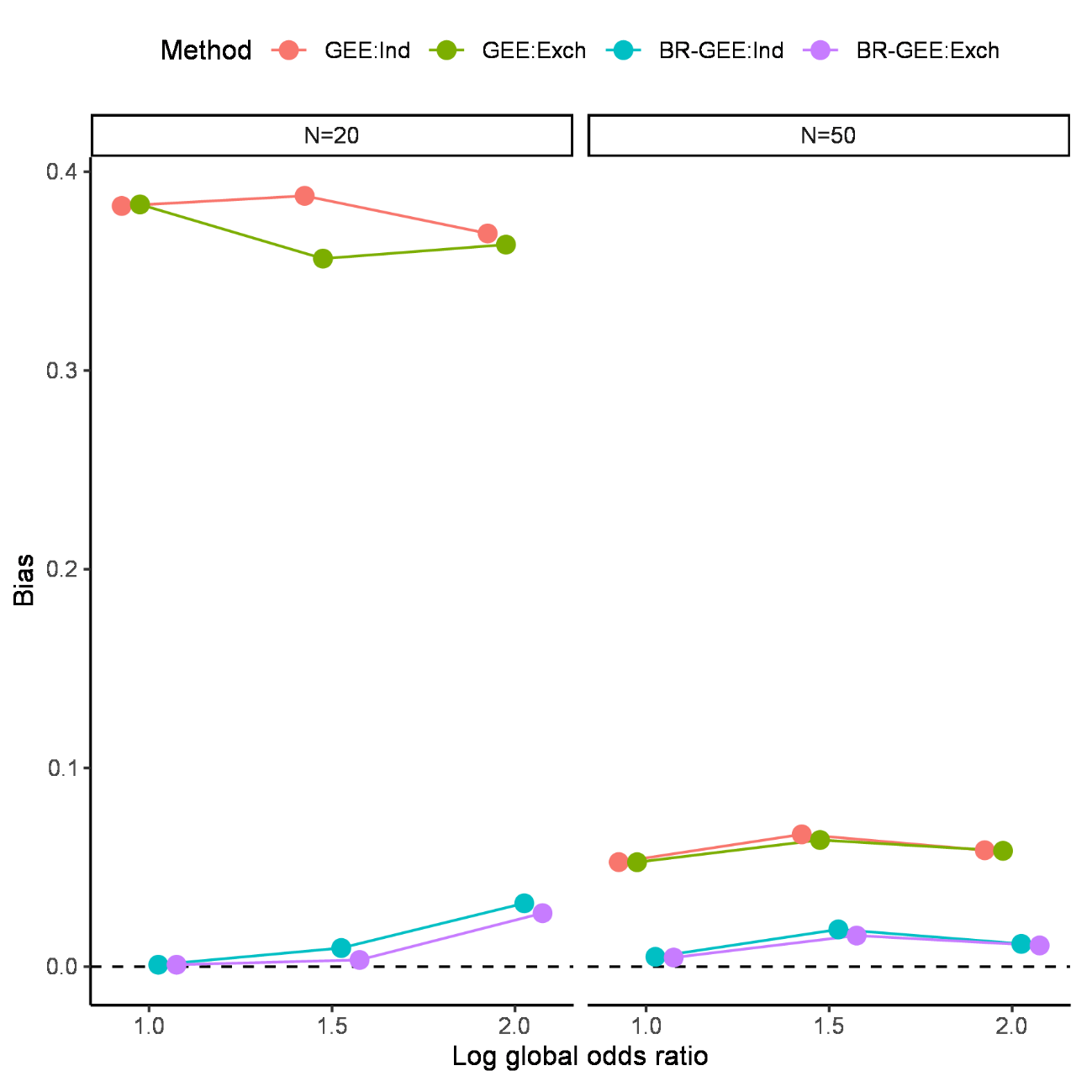


Fig. S22 Bias associated with estimates of $\beta_{1}$ with true exchangeable covariance structure (Scenario 5, K=4 and n=6). GEE:Ind, generalized estimating equation with working independent covariance structure, GEE:Exch, generalized estimating equation with working exchangeable covariance structure, BR-GEE:Ind, bias-reduced generalized estimating equation with working independent covariance structure, BR-GEE:Exch, generalized estimating equation with working exchangeable covariance structure.


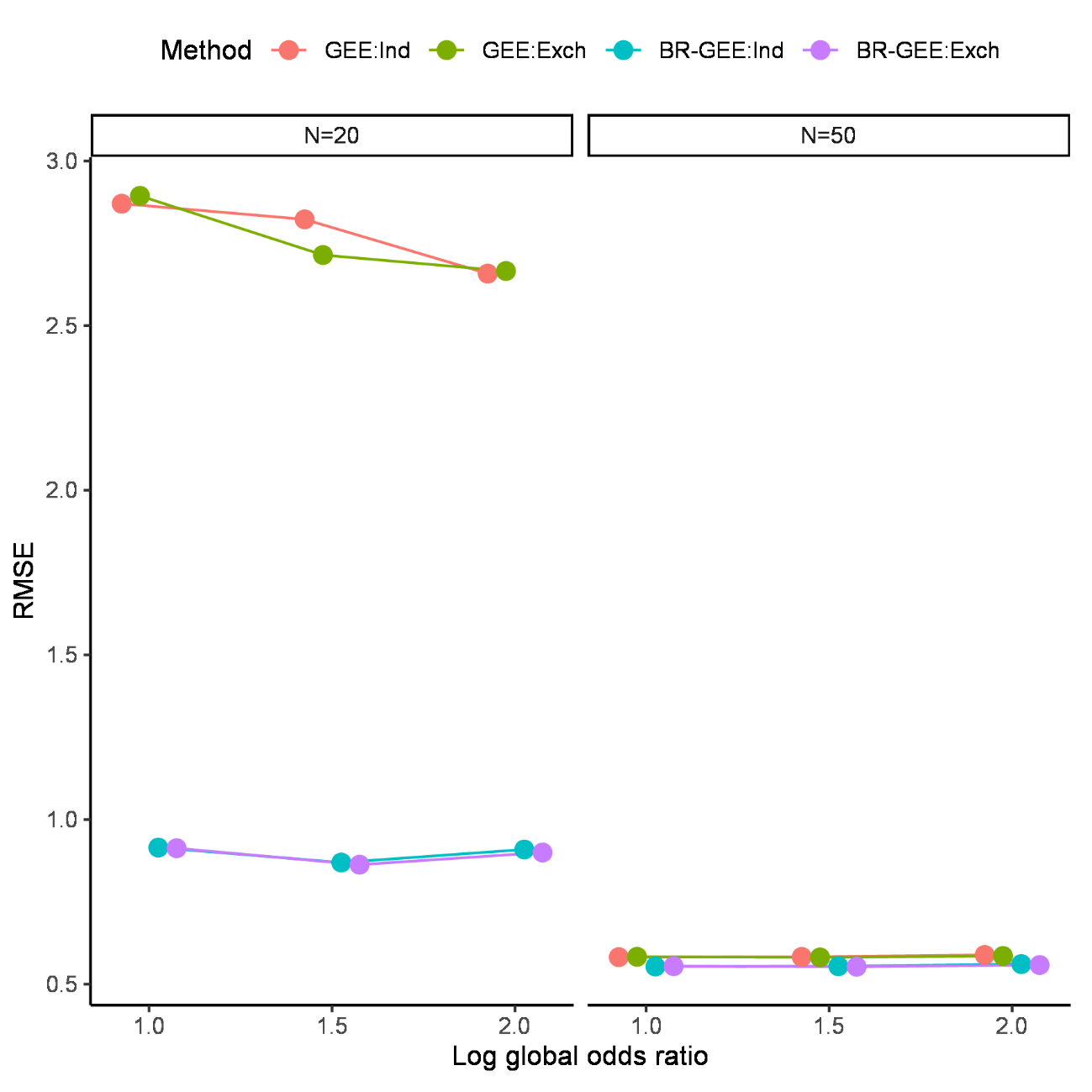


Fig. S23 RMSE associated with estimates of $\beta_{1}$ with true exchangeable covariance structure (Scenario 5, K=4 and n=6). GEE:Ind, generalized estimating equation with working independent covariance structure, GEE:Exch, generalized estimating equation with working exchangeable covariance structure, BR-GEE:Ind, bias-reduced generalized estimating equation with working independent covariance structure, BR-GEE:Exch, generalized estimating equation with working exchangeable covariance structure.


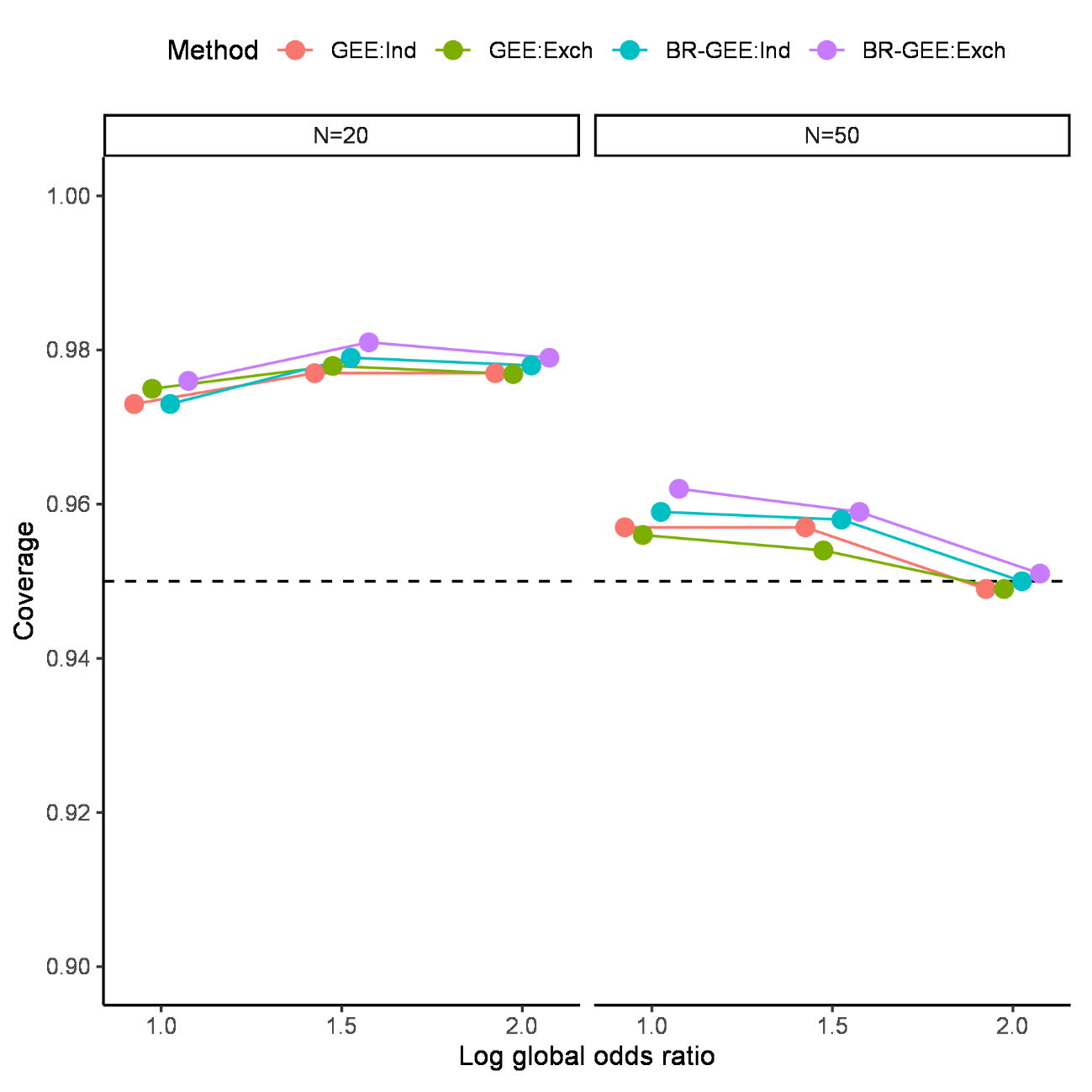


Fig. S24 95% confidence interval coverage for estimates of $\beta_{1}$ with true exchangeable covariance structure (Scenario 5, K=4 and n=6). GEE:Ind, generalized estimating equation with working independent covariance structure, GEE:Exch, generalized estimating equation with working exchangeable covariance structure, BR-GEE:Ind, bias-reduced generalized estimating equation with working independent covariance structure, BR-GEE:Exch, generalized estimating equation with working exchangeable covariance structure.


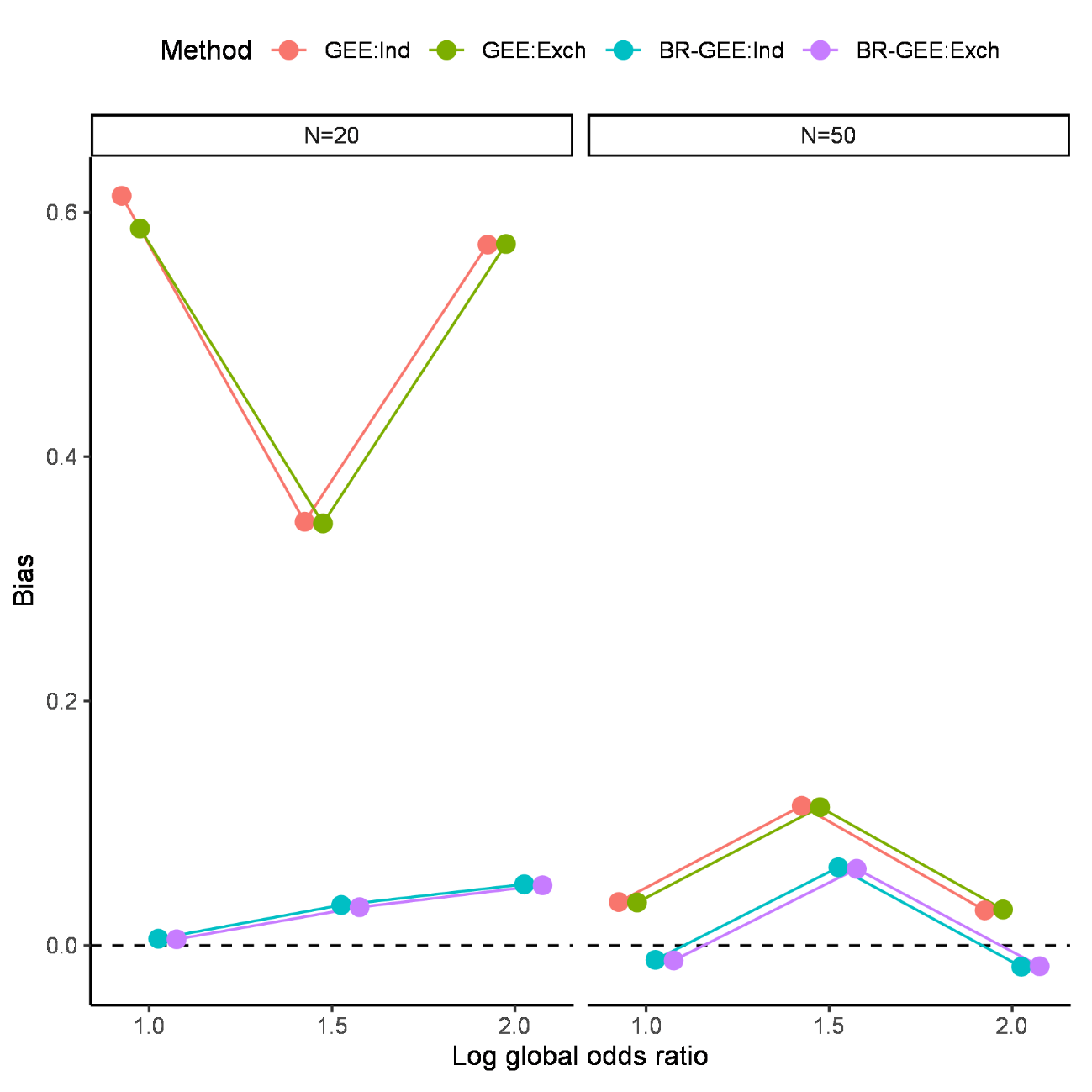


Fig. S25 Bias associated with estimates of $\beta_{1}$ with true AR-type covariance structure (Scenario 5, K=4 and n=6). GEE:Ind, generalized estimating equation with working independent covariance structure, GEE:Exch, generalized estimating equation with working exchangeable covariance structure, BR-GEE:Ind, bias-reduced generalized estimating equation with working independent covariance structure, BR-GEE:Exch, generalized estimating equation with working exchangeable covariance structure.


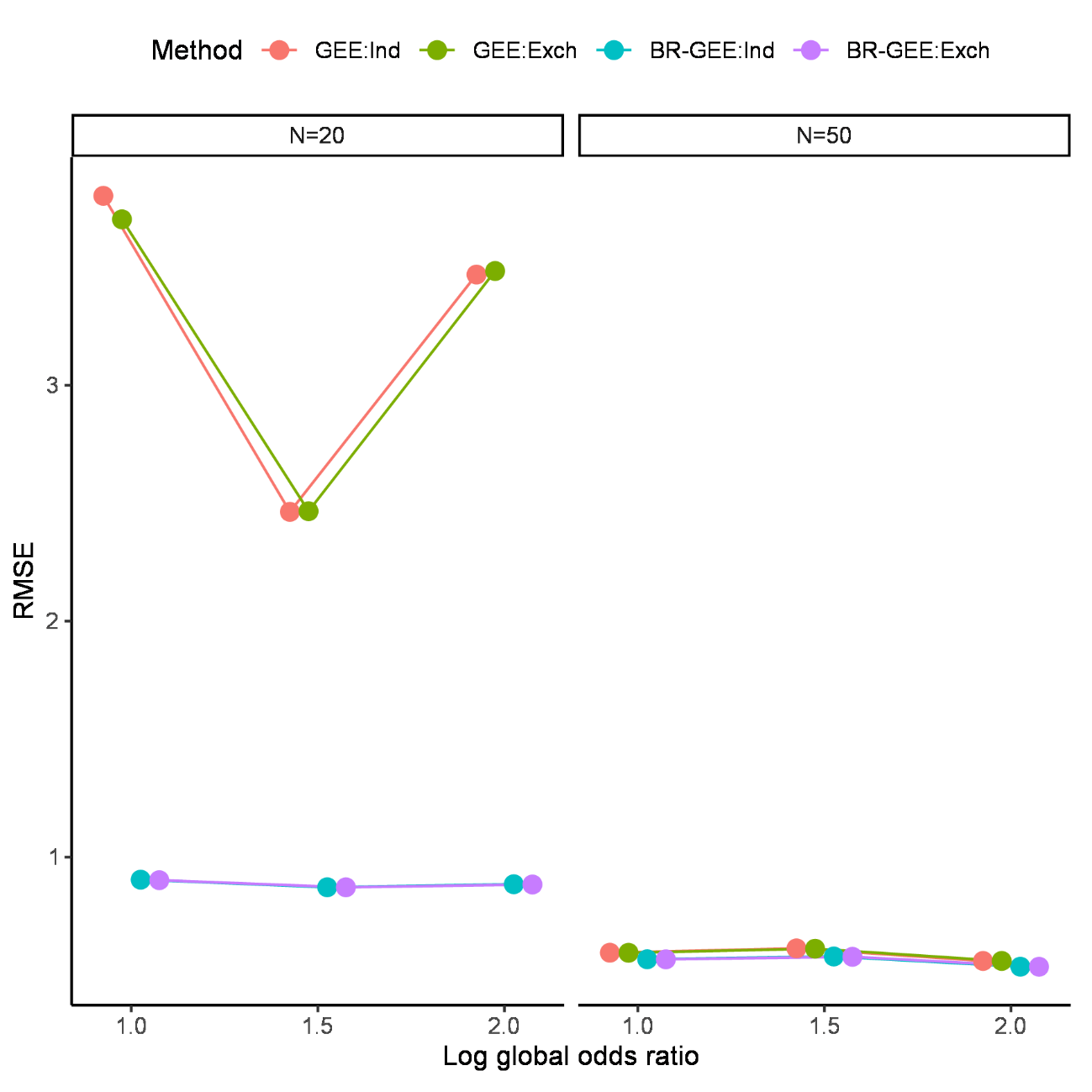


Fig. S26 RMSE associated with estimates of $\beta_{1}$ with true AR-type covariance structure (Scenario 5, K=4 and n=6). GEE:Ind, generalized estimating equation with working independent covariance structure, GEE:Exch, generalized estimating equation with working exchangeable covariance structure, BR-GEE:Ind, bias-reduced generalized estimating equation with working independent covariance structure, BR-GEE:Exch, generalized estimating equation with working exchangeable covariance structure.


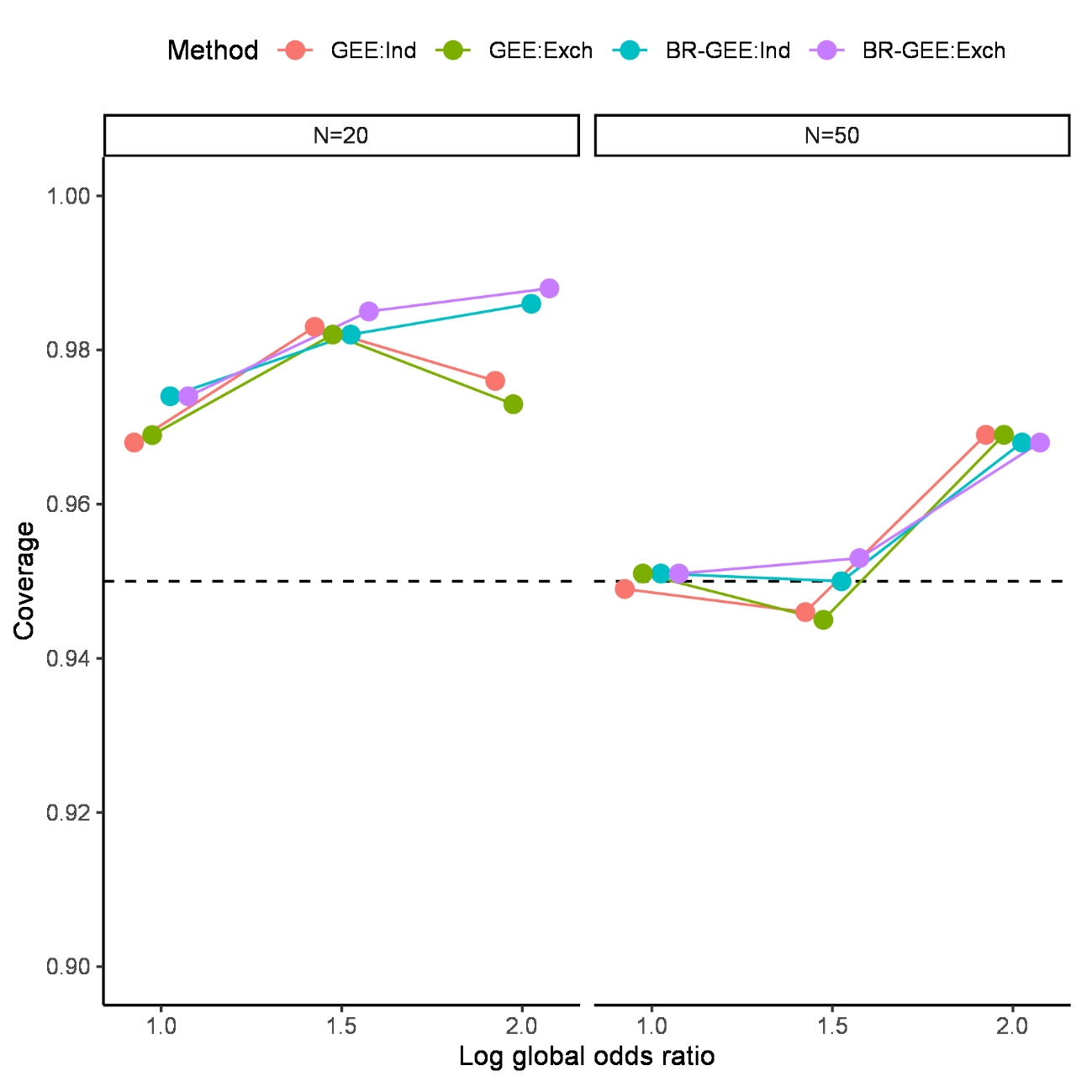


Fig. S27 95% confidence interval coverage for estimates of $\beta_{1}$ with true AR-type covariance structure (Scenario 5, K=4 and n=6). GEE:Ind, generalized estimating equation with working independent covariance structure, GEE:Exch, generalized estimating equation with working exchangeable covariance structure, BR-GEE:Ind, bias-reduced generalized estimating equation with working independent covariance structure, BR-GEE:Exch, generalized estimating equation with working exchangeable covariance structure.

# Appendix E. Coverage of 95% confidence interval by methods of standard error estimation.

The simulation results of coverage probability of the 95% confidence interval based on the Mancl and DeRouen’s bias-corrected covariance estimates in addition to $\boldsymbol{\Sigma}_{\boldsymbol{GEE}}$ and $\boldsymbol{\Sigma}_{\boldsymbol{BR-GEE}}$ assuming sample size N=20 or 50 are shown. Data generation was repeated 1000 times..

Table. S5 95% confidence interval coverage for estimates of $\beta_{1}$ with true exchangeable covariance structure (Scenario 1, K=3 and n=4).

| N | LGOR | method | 95% CI based on $\Sigma_{GEE}$ | 95% CI based on $\Sigma_{BR-GEE}$ | 95% CI based on $\Sigma_{MD}$ |
| --- | --- | --- | --- | --- | --- |
| 20 | 1.0 | GEE:Ind | 0.920 | --- | 0.927 |
|  |  | GEE:Exch | 0.920 | --- | 0.928 |
|  |  | BR-GEE:Ind | 0.939 | 0.952 | 0.949 |
|  |  | BR-GEE:Exch | 0.940 | 0.955 | 0.951 |
|  | 1.5 | GEE:Ind | 0.923 | --- | 0.929 |
|  |  | GEE:Exch | 0.923 | --- | 0.931 |
|  |  | BR-GEE:Ind | 0.940 | 0.951 | 0.949 |
|  |  | BR-GEE:Exch | 0.938 | 0.955 | 0.948 |
|  | 2.0 | GEE:Ind | 0.923 | --- | 0.933 |
|  |  | GEE:Exch | 0.927 | --- | 0.935 |
|  |  | BR-GEE:Ind | 0.941 | 0.953 | 0.955 |
|  |  | BR-GEE:Exch | 0.943 | 0.960 | 0.963 |
| 50 | 1.0 | GEE:Ind | 0.961 | --- | 0.967 |
|  |  | GEE:Exch | 0.960 | --- | 0.968 |
|  |  | BR-GEE:Ind | 0.963 | 0.960 | 0.968 |
|  |  | BR-GEE:Exch | 0.963 | 0.961 | 0.969 |
|  | 1.5 | GEE:Ind | 0.968 | --- | 0.973 |
|  |  | GEE:Exch | 0.966 | --- | 0.975 |
|  |  | BR-GEE:Ind | 0.964 | 0.960 | 0.972 |
|  |  | BR-GEE:Exch | 0.967 | 0.964 | 0.972 |
|  | 2.0 | GEE:Ind | 0.966 | --- | 0.974 |
|  |  | GEE:Exch | 0.970 | --- | 0.978 |
|  |  | BR-GEE:Ind | 0.968 | 0.965 | 0.974 |
|  |  | BR-GEE:Exch | 0.969 | 0.969 | 0.977 |

LGOR: log global odds ratio, 95%CI: 95% confidence interval, GEE:Ind, generalized estimating equation with working independent covariance structure, GEE:Exch, generalized estimating equation with working exchangeable covariance structure, BR-GEE:Ind, bias-reduced generalized estimating equation with working independent covariance structure, BR-GEE:Exch, generalized estimating equation with working exchangeable covariance structure.

Table. S6 95% confidence interval coverage for estimates of $\beta_{1}$ with true AR-type covariance structure (Scenario 1, K=3 and n=4).

| N | LGOR | method | 95% CI based on $\Sigma_{GEE}$ | 95% CI based on $\Sigma_{BR-GEE}$ | 95% CI based on $\Sigma_{MD}$ |
| --- | --- | --- | --- | --- | --- |
| 20 | 1.0 | GEE:Ind | 0.925 | --- | 0.933 |
|  |  | GEE:Exch | 0.925 | --- | 0.933 |
|  |  | BR-GEE:Ind | 0.941 | 0.959 | 0.955 |
|  |  | BR-GEE:Exch | 0.941 | 0.958 | 0.955 |
|  | 1.5 | GEE:Ind | 0.934 | --- | 0.937 |
|  |  | GEE:Exch | 0.933 | --- | 0.937 |
|  |  | BR-GEE:Ind | 0.960 | 0.971 | 0.966 |
|  |  | BR-GEE:Exch | 0.960 | 0.974 | 0.965 |
|  | 2.0 | GEE:Ind | 0.925 | --- | 0.932 |
|  |  | GEE:Exch | 0.925 | --- | 0.932 |
|  |  | BR-GEE:Ind | 0.937 | 0.959 | 0.947 |
|  |  | BR-GEE:Exch | 0.937 | 0.963 | 0.949 |
| 50 | 1.0 | GEE:Ind | 0.961 | --- | 0.963 |
|  |  | GEE:Exch | 0.959 | --- | 0.963 |
|  |  | BR-GEE:Ind | 0.957 | 0.953 | 0.968 |
|  |  | BR-GEE:Exch | 0.956 | 0.952 | 0.968 |
|  | 1.5 | GEE:Ind | 0.963 | --- | 0.973 |
|  |  | GEE:Exch | 0.964 | --- | 0.972 |
|  |  | BR-GEE:Ind | 0.966 | 0.964 | 0.972 |
|  |  | BR-GEE:Exch | 0.965 | 0.964 | 0.972 |
|  | 2.0 | GEE:Ind | 0.955 | --- | 0.962 |
|  |  | GEE:Exch | 0.951 | --- | 0.961 |
|  |  | BR-GEE:Ind | 0.952 | 0.951 | 0.958 |
|  |  | BR-GEE:Exch | 0.954 | 0.953 | 0.960 |

LGOR: log global odds ratio, 95%CI: 95% confidence interval, GEE:Ind, generalized estimating equation with working independent covariance structure, GEE:Exch, generalized estimating equation with working exchangeable covariance structure, BR-GEE:Ind, bias-reduced generalized estimating equation with working independent covariance structure, BR-GEE:Exch, generalized estimating equation with working exchangeable covariance structure.

Table. S7 95% confidence interval coverage for estimates of $\beta_{1}$ with true exchangeable covariance structure (Scenario 3, K=3 and n=6).

| N | LGOR | method | 95% CI based on $\Sigma_{GEE}$ | 95% CI based on $\Sigma_{BR-GEE}$ | 95% CI based on $\Sigma_{MD}$ |
| --- | --- | --- | --- | --- | --- |
| 20 | 1.0 | GEE:Ind | 0.927 | --- | 0.929 |
|  |  | GEE:Exch | 0.929 | --- | 0.932 |
|  |  | BR-GEE:Ind | 0.948 | 0.966 | 0.952 |
|  |  | BR-GEE:Exch | 0.946 | 0.970 | 0.956 |
|  | 1.5 | GEE:Ind | 0.927 | --- | 0.934 |
|  |  | GEE:Exch | 0.930 | --- | 0.937 |
|  |  | BR-GEE:Ind | 0.946 | 0.967 | 0.956 |
|  |  | BR-GEE:Exch | 0.950 | 0.969 | 0.961 |
|  | 2.0 | GEE:Ind | 0.938 | --- | 0.943 |
|  |  | GEE:Exch | 0.943 | --- | 0.947 |
|  |  | BR-GEE:Ind | 0.948 | 0.966 | 0.959 |
|  |  | BR-GEE:Exch | 0.957 | 0.971 | 0.965 |
| 50 | 1.0 | GEE:Ind | 0.961 | --- | 0.971 |
|  |  | GEE:Exch | 0.963 | --- | 0.973 |
|  |  | BR-GEE:Ind | 0.963 | 0.955 | 0.968 |
|  |  | BR-GEE:Exch | 0.964 | 0.960 | 0.969 |
|  | 1.5 | GEE:Ind | 0.961 | --- | 0.965 |
|  |  | GEE:Exch | 0.962 | --- | 0.967 |
|  |  | BR-GEE:Ind | 0.961 | 0.958 | 0.965 |
|  |  | BR-GEE:Exch | 0.961 | 0.960 | 0.966 |
|  | 2.0 | GEE:Ind | 0.961 | --- | 0.974 |
|  |  | GEE:Exch | 0.960 | --- | 0.970 |
|  |  | BR-GEE:Ind | 0.962 | 0.956 | 0.971 |
|  |  | BR-GEE:Exch | 0.961 | 0.957 | 0.970 |

LGOR: log global odds ratio, 95%CI: 95% confidence interval, GEE:Ind, generalized estimating equation with working independent covariance structure, GEE:Exch, generalized estimating equation with working exchangeable covariance structure, BR-GEE:Ind, bias-reduced generalized estimating equation with working independent covariance structure, BR-GEE:Exch, generalized estimating equation with working exchangeable covariance structure.

Table. S8 95% confidence interval coverage for estimates of $\beta_{1}$ with true AR-type covariance structure (Scenario 3, K=3 and n=6).

| N | LGOR | method | 95% CI based on $\Sigma_{GEE}$ | 95% CI based on $\Sigma_{BR-GEE}$ | 95% CI based on $\Sigma_{MD}$ |
| --- | --- | --- | --- | --- | --- |
| 20 | 1.0 | GEE:Ind | 0.936 | --- | 0.940 |
|  |  | GEE:Exch | 0.936 | --- | 0.940 |
|  |  | BR-GEE:Ind | 0.953 | 0.972 | 0.962 |
|  |  | BR-GEE:Exch | 0.952 | 0.971 | 0.963 |
|  | 1.5 | GEE:Ind | 0.938 | --- | 0.944 |
|  |  | GEE:Exch | 0.939 | --- | 0.944 |
|  |  | BR-GEE:Ind | 0.951 | 0.967 | 0.964 |
|  |  | BR-GEE:Exch | 0.951 | 0.968 | 0.965 |
|  | 2.0 | GEE:Ind | 0.934 | --- | 0.942 |
|  |  | GEE:Exch | 0.933 | --- | 0.943 |
|  |  | BR-GEE:Ind | 0.954 | 0.970 | 0.969 |
|  |  | BR-GEE:Exch | 0.955 | 0.969 | 0.969 |
| 50 | 1.0 | GEE:Ind | 0.969 | --- | 0.976 |
|  |  | GEE:Exch | 0.968 | --- | 0.975 |
|  |  | BR-GEE:Ind | 0.969 | 0.961 | 0.974 |
|  |  | BR-GEE:Exch | 0.968 | 0.962 | 0.974 |
|  | 1.5 | GEE:Ind | 0.972 | --- | 0.977 |
|  |  | GEE:Exch | 0.967 | --- | 0.979 |
|  |  | BR-GEE:Ind | 0.971 | 0.969 | 0.975 |
|  |  | BR-GEE:Exch | 0.970 | 0.968 | 0.975 |
|  | 2.0 | GEE:Ind | 0.969 | --- | 0.974 |
|  |  | GEE:Exch | 0.967 | --- | 0.975 |
|  |  | BR-GEE:Ind | 0.966 | 0.965 | 0.975 |
|  |  | BR-GEE:Exch | 0.964 | 0.963 | 0.975 |

LGOR: log global odds ratio, 95%CI: 95% confidence interval, GEE:Ind, generalized estimating equation with working independent covariance structure, GEE:Exch, generalized estimating equation with working exchangeable covariance structure, BR-GEE:Ind, bias-reduced generalized estimating equation with working independent covariance structure, BR-GEE:Exch, generalized estimating equation with working exchangeable covariance structure.

Table. S9 95% confidence interval coverage for estimates of $\beta_{1}$ with true exchangeable covariance structure (Scenario 4, K=4 and n=4).

| N | LGOR | method | 95% CI based on $\Sigma_{GEE}$ | 95% CI based on $\Sigma_{BR-GEE}$ | 95% CI based on $\Sigma_{MD}$ |
| --- | --- | --- | --- | --- | --- |
| 20 | 1.0 | GEE:Ind | 0.965 | --- | 0.975 |
|  |  | GEE:Exch | 0.966 | --- | 0.973 |
|  |  | BR-GEE:Ind | 0.966 | 0.969 | 0.977 |
|  |  | BR-GEE:Exch | 0.967 | 0.969 | 0.976 |
|  | 1.5 | GEE:Ind | 0.973 | --- | 0.983 |
|  |  | GEE:Exch | 0.972 | --- | 0.983 |
|  |  | BR-GEE:Ind | 0.974 | 0.975 | 0.985 |
|  |  | BR-GEE:Exch | 0.974 | 0.977 | 0.985 |
|  | 2.0 | GEE:Ind | 0.967 | --- | 0.981 |
|  |  | GEE:Exch | 0.972 | --- | 0.984 |
|  |  | BR-GEE:Ind | 0.971 | 0.972 | 0.981 |
|  |  | BR-GEE:Exch | 0.975 | 0.975 | 0.984 |
| 50 | 1.0 | GEE:Ind | 0.958 | --- | 0.967 |
|  |  | GEE:Exch | 0.960 | --- | 0.966 |
|  |  | BR-GEE:Ind | 0.961 | 0.958 | 0.971 |
|  |  | BR-GEE:Exch | 0.961 | 0.960 | 0.969 |
|  | 1.5 | GEE:Ind | 0.945 | --- | 0.957 |
|  |  | GEE:Exch | 0.947 | --- | 0.955 |
|  |  | BR-GEE:Ind | 0.951 | 0.949 | 0.956 |
|  |  | BR-GEE:Exch | 0.952 | 0.953 | 0.959 |
|  | 2.0 | GEE:Ind | 0.949 | --- | 0.955 |
|  |  | GEE:Exch | 0.951 | --- | 0.956 |
|  |  | BR-GEE:Ind | 0.953 | 0.949 | 0.959 |
|  |  | BR-GEE:Exch | 0.950 | 0.950 | 0.960 |

LGOR: log global odds ratio, 95%CI: 95% confidence interval, GEE:Ind, generalized estimating equation with working independent covariance structure, GEE:Exch, generalized estimating equation with working exchangeable covariance structure, BR-GEE:Ind, bias-reduced generalized estimating equation with working independent covariance structure, BR-GEE:Exch, generalized estimating equation with working exchangeable covariance structure.

Table. S10 95% confidence interval coverage for estimates of $\beta_{1}$ with true AR-type covariance structure (Scenario 4, K=4 and n=4).

| N | LGOR | method | 95% CI based on $\Sigma_{GEE}$ | 95% CI based on $\Sigma_{BR-GEE}$ | 95% CI based on $\Sigma_{MD}$ |
| --- | --- | --- | --- | --- | --- |
| 20 | 1.0 | GEE:Ind | 0.956 | --- | 0.970 |
|  |  | GEE:Exch | 0.955 | --- | 0.970 |
|  |  | BR-GEE:Ind | 0.961 | 0.959 | 0.973 |
|  |  | BR-GEE:Exch | 0.959 | 0.957 | 0.973 |
|  | 1.5 | GEE:Ind | 0.965 | --- | 0.971 |
|  |  | GEE:Exch | 0.966 | --- | 0.970 |
|  |  | BR-GEE:Ind | 0.966 | 0.967 | 0.974 |
|  |  | BR-GEE:Exch | 0.966 | 0.968 | 0.974 |
|  | 2.0 | GEE:Ind | 0.970 | --- | 0.975 |
|  |  | GEE:Exch | 0.971 | --- | 0.976 |
|  |  | BR-GEE:Ind | 0.973 | 0.970 | 0.978 |
|  |  | BR-GEE:Exch | 0.971 | 0.971 | 0.978 |
| 50 | 1.0 | GEE:Ind | 0.952 | --- | 0.960 |
|  |  | GEE:Exch | 0.952 | --- | 0.960 |
|  |  | BR-GEE:Ind | 0.953 | 0.952 | 0.963 |
|  |  | BR-GEE:Exch | 0.953 | 0.953 | 0.965 |
|  | 1.5 | GEE:Ind | 0.946 | --- | 0.957 |
|  |  | GEE:Exch | 0.944 | --- | 0.957 |
|  |  | BR-GEE:Ind | 0.952 | 0.949 | 0.963 |
|  |  | BR-GEE:Exch | 0.951 | 0.950 | 0.965 |
|  | 2.0 | GEE:Ind | 0.943 | --- | 0.949 |
|  |  | GEE:Exch | 0.941 | --- | 0.950 |
|  |  | BR-GEE:Ind | 0.943 | 0.942 | 0.954 |
|  |  | BR-GEE:Exch | 0.947 | 0.946 | 0.957 |

LGOR: log global odds ratio, 95%CI: 95% confidence interval, GEE:Ind, generalized estimating equation with working independent covariance structure, GEE:Exch, generalized estimating equation with working exchangeable covariance structure, BR-GEE:Ind, bias-reduced generalized estimating equation with working independent covariance structure, BR-GEE:Exch, generalized estimating equation with working exchangeable covariance structure.

Table. S11 95% confidence interval coverage for estimates of $\beta_{1}$ with true exchangeable covariance structure (Scenario 5, K=4 and n=6).

| N | LGOR | method | 95% CI based on $\Sigma_{GEE}$ | 95% CI based on $\Sigma_{BR-GEE}$ | 95% CI based on $\Sigma_{MD}$ |
| --- | --- | --- | --- | --- | --- |
| 20 | 1.0 | GEE:Ind | 0.973 | --- | 0.981 |
|  |  | GEE:Exch | 0.975 | --- | 0.979 |
|  |  | BR-GEE:Ind | 0.974 | 0.973 | 0.980 |
|  |  | BR-GEE:Exch | 0.974 | 0.976 | 0.980 |
|  | 1.5 | GEE:Ind | 0.977 | --- | 0.985 |
|  |  | GEE:Exch | 0.978 | --- | 0.985 |
|  |  | BR-GEE:Ind | 0.978 | 0.979 | 0.987 |
|  |  | BR-GEE:Exch | 0.979 | 0.981 | 0.987 |
|  | 2.0 | GEE:Ind | 0.977 | --- | 0.982 |
|  |  | GEE:Exch | 0.977 | --- | 0.983 |
|  |  | BR-GEE:Ind | 0.979 | 0.978 | 0.983 |
|  |  | BR-GEE:Exch | 0.978 | 0.979 | 0.985 |
| 50 | 1.0 | GEE:Ind | 0.957 | --- | 0.965 |
|  |  | GEE:Exch | 0.956 | --- | 0.967 |
|  |  | BR-GEE:Ind | 0.963 | 0.959 | 0.971 |
|  |  | BR-GEE:Exch | 0.963 | 0.962 | 0.969 |
|  | 1.5 | GEE:Ind | 0.957 | --- | 0.965 |
|  |  | GEE:Exch | 0.954 | --- | 0.965 |
|  |  | BR-GEE:Ind | 0.961 | 0.958 | 0.966 |
|  |  | BR-GEE:Exch | 0.960 | 0.959 | 0.964 |
|  | 2.0 | GEE:Ind | 0.949 | --- | 0.959 |
|  |  | GEE:Exch | 0.949 | --- | 0.962 |
|  |  | BR-GEE:Ind | 0.953 | 0.950 | 0.959 |
|  |  | BR-GEE:Exch | 0.951 | 0.951 | 0.960 |

LGOR: log global odds ratio, 95%CI: 95% confidence interval, GEE:Ind, generalized estimating equation with working independent covariance structure, GEE:Exch, generalized estimating equation with working exchangeable covariance structure, BR-GEE:Ind, bias-reduced generalized estimating equation with working independent covariance structure, BR-GEE:Exch, generalized estimating equation with working exchangeable covariance structure.

Table. S12 95% confidence interval coverage for estimates of $\beta_{1}$ with true AR-type covariance structure (Scenario 5, K=4 and n=6).

| N | LGOR | method | 95% CI based on $\Sigma_{GEE}$ | 95% CI based on $\Sigma_{BR-GEE}$ | 95% CI based on $\Sigma_{MD}$ |
| --- | --- | --- | --- | --- | --- |
| 20 | 1.0 | GEE:Ind | 0.968 | --- | 0.975 |
|  |  | GEE:Exch | 0.969 | --- | 0.976 |
|  |  | BR-GEE:Ind | 0.975 | 0.974 | 0.980 |
|  |  | BR-GEE:Exch | 0.975 | 0.974 | 0.981 |
|  | 1.5 | GEE:Ind | 0.983 | --- | 0.988 |
|  |  | GEE:Exch | 0.982 | --- | 0.987 |
|  |  | BR-GEE:Ind | 0.986 | 0.982 | 0.990 |
|  |  | BR-GEE:Exch | 0.985 | 0.985 | 0.990 |
|  | 2.0 | GEE:Ind | 0.976 | --- | 0.981 |
|  |  | GEE:Exch | 0.973 | --- | 0.981 |
|  |  | BR-GEE:Ind | 0.978 | 0.986 | 0.982 |
|  |  | BR-GEE:Exch | 0.979 | 0.988 | 0.983 |
| 50 | 1.0 | GEE:Ind | 0.949 | --- | 0.961 |
|  |  | GEE:Exch | 0.951 | --- | 0.960 |
|  |  | BR-GEE:Ind | 0.955 | 0.951 | 0.964 |
|  |  | BR-GEE:Exch | 0.954 | 0.951 | 0.964 |
|  | 1.5 | GEE:Ind | 0.946 | --- | 0.960 |
|  |  | GEE:Exch | 0.945 | --- | 0.958 |
|  |  | BR-GEE:Ind | 0.951 | 0.950 | 0.960 |
|  |  | BR-GEE:Exch | 0.953 | 0.953 | 0.962 |
|  | 2.0 | GEE:Ind | 0.969 | --- | 0.975 |
|  |  | GEE:Exch | 0.969 | --- | 0.976 |
|  |  | BR-GEE:Ind | 0.969 | 0.968 | 0.975 |
|  |  | BR-GEE:Exch | 0.968 | 0.968 | 0.973 |

LGOR: log global odds ratio, 95%CI: 95% confidence interval, GEE:Ind, generalized estimating equation with working independent covariance structure, GEE:Exch, generalized estimating equation with working exchangeable covariance structure, BR-GEE:Ind, bias-reduced generalized estimating equation with working independent covariance structure, BR-GEE:Exch, generalized estimating equation with working exchangeable covariance structure.
